# First Detection and Identification of Southern Tomato Virus Infecting Tomatoes in Oklahoma with Complete Genome Characterization and Insights into Global Genetic Diversity

**DOI:** 10.3390/v17091193

**Published:** 2025-08-30

**Authors:** Salil Jindal, Akhtar Ali

**Affiliations:** Department of Biological Science, Oxley College of Health & Natural Sciences, The University of Tulsa, Tulsa, OK 74104, USA; saj1129@utulsa.edu

**Keywords:** high-throughput sequencing, phylogenetics, molecular evolution, genetic diversity, recombination, purifying selection, protein disorder southern tomato virus

## Abstract

Southern tomato virus (STV) or *Amalgavirus lycopersici* is a persistent virus impacting tomato crops globally. This study identified new STV isolates from Oklahoma and analyzed their evolutionary relationship to global STV isolates. Phylogenetic analyses (complete genomes or individual genes) grouped STV isolates into two distinct clades, independent of geographic origin or host. Notably, Oklahoma isolates formed a separate cluster from previously reported isolates in the United States of America (USA). Coalescent analysis suggested the most recent common ancestor of STV fusion protein emerged around 135 years ago. Genetic diversity among STV isolates was low, with slightly more variability in the RNA-dependent RNA polymerase (RdRp) gene than the p42 gene. Both genes showed strong purifying selection. No recombination events were detected across complete genomes. Structure analysis revealed that the p42 protein, particularly its C-terminal region, displayed higher disorder, indicating a possible role in host interactions and viral adaptability. These findings deepen our understanding of STV’s evolution and highlight the need for ongoing surveillance and broader genomic sampling.

## 1. Introduction

Tomato (*Solanum lycopersicum* L.) is among the most cultivated solanaceous vegetables having its origin in western South America [1]. It is consumed both fresh and in processed forms such as soups, sauces, and ketchup [2]. In addition to its culinary uses, tomatoes are a valuable source for secondary metabolites with antioxidant properties and potential anti-cancer benefits [3]. In the early 2020s, global tomato production was reported at approximately 186.82 million tons, cultivated over 5 million hectares with an average productivity of 36.97 tons/hectare [4]. Despite its economic and nutritional importance, tomato production is significantly hindered by its high susceptibility to viral diseases, which can result in yield losses ranging from 70 to 95%, thereby impacting market availability [5].

Southern tomato virus (STV; *Amalgavirus lycopersici*) belongs to the family *Amalgaviridae*. It was initially identified in symptomatic tomato plants exhibiting stunting, fruit discoloration, and size reduction in California and Mississippi (USA) as well as in southwestern Mexico [6]. Over the past decade, STV has been reported in tomato crops across several countries, including Albania [7], Bangladesh [8], China [9,10,11], Colombia [12], France [13], Germany [14], Greece [15], Italy [16], Korea [17], Panama [18], Pakistan [11], Serbia [19], Spain [20], Turkey [21], and United Kingdom [22]. The STV genome consists of a monopartite double stranded RNA (dsRNA) molecule approximately 3.5 kb in length, containing two overlapping open-reading frames (ORFs). ORF 1 encodes a putative coat protein (p42), while ORF 2 encodes a fusion protein with RNA-dependent RNA polymerase: (RdRp) activity, expressed via a +1 ribosomal frameshift. STV is efficiently transmitted through seeds but not by mechanical means or grafting [6]. Notably, STV is classified as a cryptic plant virus, often causing no visible symptoms in singly infected plants [11].

The cryptic nature of STV presents significant challenges in elucidating its genetic diversity and evolutionary dynamics factors that are critical for understanding its epidemiology [23]. This ambiguity also hampers the development of reliable diagnostic tools and the implementation of effective disease management strategies [24]. Previous investigations into STV population dynamics have been limited, with studies focusing on a small number of complete genome sequences [11,18,21] or targeting only the putative coat protein gene [25]. In contrast, the present study utilizes over 100 STV isolates available in the GenBank database to conduct a comprehensive analysis of the virus’s genetic diversity and evolutionary patterns. Additionally, this study reports, for the first time, the detection and characterization of STV isolates from tomatoes in Oklahoma. The extensive analysis conducted here offers new insights into STV population structure. Furthermore, this study employs, for the first time, advanced Bayesian evolutionary analysis using BEAST platform, as well as protein disorder prediction, to explore the evolutionary and functional characteristics of STV in greater depth.

## 2. Materials and Methods

### 2.1. Sample Collection and Total RNA Extraction

In 2023, tomato leaf samples exhibiting typical virus-like symptoms were collected from five plants (designated K71 to K75) in Cherokee County, Oklahoma. Total RNA was extracted using the TRI-Reagent protocol (Molecular Research Center, Cincinnati, OH, USA) and quantified with a NanoDrop spectrophotometer (Thermo Fisher Scientific; Wilmington, DE, USA).

### 2.2. High Throughput Sequencing and RT-PCR Confirmation

Total RNA extracted from the five samples was combined into two pooled composite samples. RNA from samples K71 and K72 was normalized and pooled to generate composite sample OK1, while RNA from samples K73, K74, and K75 was pooled to create composite sample OK2. These pooled samples were subjected to high-throughput sequencing (HTS) using the NextSeq 500/550 High-Output Kit v2.5 (Illumina, San Diego, CA, USA) at the Genomics Facility, Oklahoma State University (Stillwater, OK, USA). Paired-end reads generated from HTS were trimmed and assembled de novo using CLC Genomics Workbench v22.0.1 (Qiagen, CLC bio, Aarhus, Denmark). The resulting assembled sequences were analyzed using BLASTn and BLASTx (NCBI, Bethesda, MD, USA) against the non-redundant GenBank database (NCBI, Bethesda, MD, USA) for sequence identification.

Total RNA from individual samples was reverse transcribed into complementary DNA (cDNA) using random hexamers and MMLV reverse transcriptase, following the manufacturer’s protocols (Genscript, Piscataway, NJ, USA). Reverse transcription PCR (RT-PCR) was performed in a 20 µL reaction containing 1 µL of cDNA template, Taq DNA polymerase (Genscript, Piscataway NJ, USA), 10x Taq Buffer, 2 mM dNTPs, RNase A, and sequence specific primers (Table 1). Amplified PCR products were visualized on a 1% TBE agarose gel and purified using the EZNA Cycle Pure Kit (Omega Bio-Tek, Norcross, Georgia, USA). Purified products were then subjected to Sanger sequencing using Eurofins sequencing service (Eurofins-MWG, Louisville, KY, USA).

### 2.3. Multiple Sequence Alignment and Phylogenetic Analysis

Multiple sequence alignments were generated for 108 complete genomes of STV isolates (two isolates were sequenced in this study, while 106 sequences were retrieved from GenBank, last accessed on 24 June 2025) using the MUSCLE algorithm implemented in MEGA version 12 [26]. Similarly, alignments were also performed for the RdRp (108 sequences), fusion protein (108 sequences), and p42 (130 sequences). All alignments underwent pre-processing prior to downstream analysis. The best-fit nucleotide substitution model for each dataset was determined based on the lowest Bayesian Information Criterion (BIC) score using MEGA v12. For the complete genome, a Hasegawa–Kishino–Yano (HKY) model incorporating a gamma-distributed rate variation among sites and a proportion of invariant sites (HKY+G+I) was selected, and for the p42, RdRp, and fusion protein datasets, a HKY model with a parameter for invariant sites (HKY+I) was selected. Phylogenetic trees were constructed using the Maximum likelihood (ML) method in MEGA v12 with 1000 bootstrap replications. Bootstrap values exceeding 70 were considered statistically significant.

### 2.4. Analysis of Genetic Diversity and Population Structure of STV

Nucleotide diversity (π), defined as the average number of nucleotide differences per site, was estimated for complete STV genomes using DnaSP v6.12.03 [27]. A sliding window analysis was conducted across the entire genome, employing a window size of 100 nucleotides and a step size of 10 nucleotides. Additionally, population genetic parameters were calculated for the complete genomes as well as for the p42, RdRp, and fusion protein gene regions. These parameters included the number of haplotypes (H), haplotype diversity (Hd), number of segregating sites (S), total number of mutations (Eta), average number of nucleotide differences (k), and Watterson’s theta per sequence and per site (θ), along with neutrality tests such as Tajima’s D and Fu and Li’s D* statistic.

Genetic differentiation between two populations, delineated as Clade I and Clade II based on phylogenetic analysis, was evaluated for the RdRp and p42 genes using Ks, Kst*,

Z*, and Snn statistics. Statistical significance was determined through permutation testing with 1000 replicates. To further quantify the extent of genetic divergence and gene flow, fixation index (Fst) and the number of migrants per generation (Nm) were calculated. Fst values range from 0 (no differentiation) to 1 (complete differentiation), with values exceeding 0.33 indicative of limited genetic exchange and pronounced population structure [28,29,30]. Nm provides an estimate of effective gene flow between populations; values below 1 suggest restricted migration and a greater influence of genetic drift, whereas values above 1 imply sufficient gene flow to mitigate drift-driven differentiation [31].

### 2.5. Recombination Detection and Pairwise Identity Analysis

To detect potential recombination events in STV, two complementary approaches were used. First, multiple sequence alignments of 108 complete genome sequences were examined using the Recombination Detection Program (RDP v4.101) [32]. All seven primary recombination detection methods—RDP, GENECONV, Chimaera, MaxChi, BootScan, SiScan, and 3Seq—along with two secondary scanning methods, BootScan and SiScan, were applied under default settings, with the exception that sequences were designated as linear. Recombination events identified by at least four of these methods were considered statistically significant.

To further investigate recombination breakpoints, the Genetic Algorithm for Recombination Detection (GARD), available via the Datamonkey web server (https://www.datamonkey.org/ (accessed on 29 June 2025)) [33], was used. Additionally, pairwise identity analyses of the complete genome sequences, as well as the p42, RdRp, and fusion protein gene regions, were conducted using the Sequence Demarcation Tool (SDT) version 1.2 [34].

### 2.6. Bayesian Evolutionary Analysis Sampling Trees (BEAST) Analysis

To estimate the divergence time and nucleotide substitution rates of the fusion protein gene, RdRp, and p42 sequences of STV, Bayesian evolutionary analysis was conducted using BEAST v2.5.2 (Bayesian Evolutionary Analysis Sampling Trees) [35]. Sequences lacking collection dates were excluded resulting in a dataset of 104 sequences for the fusion protein gene and RdRp and 110 sequences for p42. The best fit substitution model for each dataset was determined based on the lowest BIC score in MEGA v12. In BEAUti v2.5.2, input parameters included tip dates derived from collection years available in NCBI metadata, the HKY substitution model, and a relaxed log-normal molecular clock. The tree prior was set to Coalescent: Bayesian Skyline with default settings. Two independent Markov Chain Monte Carlo (MCMC) runs were performed for 200 million generations each, with sampling every 20,000 generations. A 95% highest probability density (HPD) was used to assess statistical significance. Trace files from both MCMC runs were examined in Tracer v1.7.2 [36] to assess convergence and effective sample size. Posterior distributions from independent runs were combined using LogCombiner v2.5.2, discarding the first 10% of each run as burn-in. A maximum clade credibility (MCC) tree was then generated using TreeAnnotator v2.5.2. Final phylogenetic trees were visualized using FigTree v1.4.4 (http://tree.bio.ed.ac.uk/software/figtree/ (accessed on 10 July 2025)).

### 2.7. Protein Disorder Analysis

Intrinsically disordered proteins regions in plant RNA viruses have been widely predicted and experimentally validated [37,38,39,40,41]. The likelihood of intrinsic disorder in the p42, RdRp, and fusion protein of STV isolates obtained in this study was predicted using the IUPred2A webserver (http://iupred2a.elte.hu (accessed on 2 July 2025)) [42] and compared with the designated STV reference genome sequence (NC_011591). Further, the distribution of disordered amino acids was analyzed by comparing the N-terminal and C-terminal regions of the proteins. IUPred2A estimates the disorder propensity based on total pairwise interaction energy derived from amino acid compositions. To statistically evaluate difference in the proportion of disordered amino acids between the study isolates and the reference genome, the non-parametric Wilcoxon rank-sum test was performed in R with a significance threshold of α = 0.05.

### 2.8. Analysis of Selective Pressure 

To assess the selection pressure acting on the RdRp (*n* = 108), fusion protein (*n* = 108) and p42 (*n* = 130) genes of STV, analysis were conducted using Datamonkey webserver [35]. Three computational methods were employed: Fast Unconstrained Bayesian AppRoximation (FUBAR) [43], Single Likelihood Ancestor Counting (SLAC), and Mixed Effects Model of Evolution (MEME) [44].

## 3. Results

### 3.1. Genome Assembly of STV Isolates and RT-PCR Confirmation

High-throughput sequencing (HTS) of composite samples OK1 and OK2 yielded 51,084,045 and 56,242,802 trimmed reads, respectively, with an average reads length of 129.6 bp and 133.86 bp. De novo assembled contigs were subjected to BLASTn and BLASTx searches against the NCBI non-redundant GenBank database, resulting in the identification of two novel STV genomes sequences from tomatoes in Oklahoma (GenBank accession numbers: PV786594-OK1 and PV786595-OK2 isolates) exhibiting over 99% identity with the reference isolate MK948545.

To confirm the presence of STV in the individual tomato samples, RT-PCR was conducted using total RNA and STV specific primers targeting conserved genomic regions. The expected amplification products were obtained from all five symptomatic samples, while no amplification was obtained in the healthy control samples (Figures S1 and S2). PCR products were subsequently purified and sequenced via Sanger sequencing. The resulting sequences exhibited over 99% identity to known STV isolates, thereby validating the presence of STV infection in the tested samples.

### 3.2. Phylogenetic Analysis

**Figure 1:**
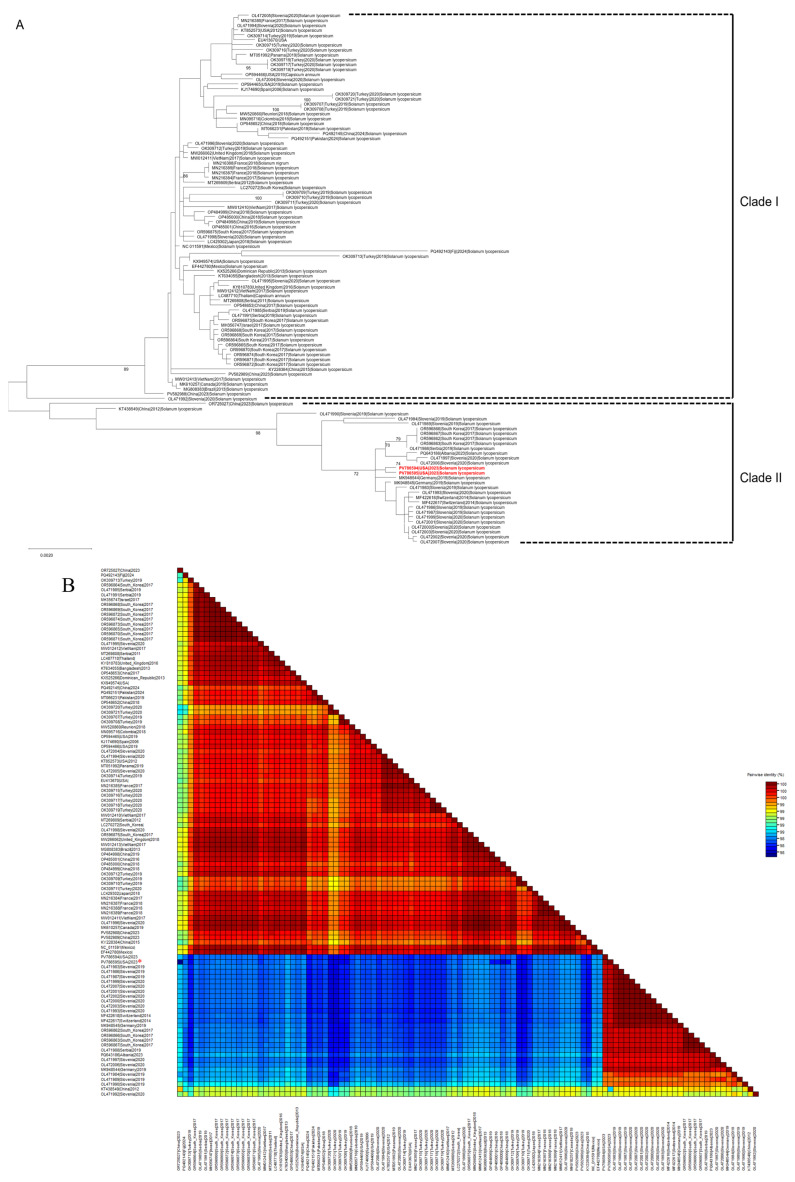
Evolutionary analysis of southern tomato virus (STV) based on 108 complete genome sequences. (**A**) Maximum likelihood phylogenetic tree with 1000 bootstrap replicates based on nucleotide sequences of complete genomes of STV, (**B**) Nucleotide pairwise identity heatmap of complete genomes of STV. (Highlighted in red color or asterisk-marked (*) STV isolates represent those identified in this study).

Based on complete genome sequences, all 108 STV isolates were clustered into two major phylogenetic clades designated Clade I and Clade II (Figure 1A). The majority of isolates (79 isolates) were grouped into Clade I and originated from diverse regions, including Asia (Bangladesh, China, Israel, Japan, Pakistan, South Korea, Thailand, Vietnam, and Turkey), Europe (France, Serbia, Slovenia, Spain, United Kingdom, and Turkey), North America (Canada, Dominican Republic, Mexico, Panama, and US), South America (Brazil and Colombia), and Oceania (Fiji). In contrast, Clade II comprised a smaller number of isolates (29 isolates), including those from Albania, Slovenia, and Switzerland, and selected isolates from China, Germany, Serbia, and South Korea. Notably, the two STV isolates characterized in this study were grouped within Clade II, whereas previously reported STV isolates from the USA were placed in Clade I.

The phylogenetic analysis of the fusion protein gene also produced a tree topology congruent with that of the complete genomes (Figure S3), with the newly characterized isolates again placed in Clade II, in contrast to previously reported USA isolates, which remained in Clade I.

Similarly, the phylogenetic tree based on the RdRp gene (Figure S4) showed a comparable topology to the complete genome tree. All isolates previously grouped in Clade II remained consistent, with the exception of OL741992 from Slovenia which was grouped in Clade I in the RdRp-based tree. The Oklahoma isolates from this study continued to cluster within Clade II.

The phylogenetic tree constructed from the p42 gene (Figure S5) largely mirrored the topology of the complete genome tree, with one exception: accession OR725027 from China, which clustered in Clade II in the complete genome tree but grouped in Clade I in the p42-based analysis. The isolates reported in this study consistently clustered in Clade II.

Pairwise nucleotide identity analysis performed using SDT for the complete genomes (Figure 1B), fusion protein (Figure S6), RdRp (Figure S7), and p42 (Figure S8) sequences further supported the clustering patterns observed in the corresponding phylogenetic analyses.

### 3.3. Genetic Diversity, Genetic Differentiation, and Migration in STV Populations

To characterize the genetic variability of STV, comprehensive population genetic analysis was performed using complete genome and gene-specific datasets. Analysis of 108 complete STV genomes revealed a relatively low mutation rate (Eta = 6.05%) but high haplotype diversity (H_d_ = 0.979) and a moderate nucleotide diversity (π) of 0.00741. Neutrality tests yielded statistically significant negative values for Fu and Li’s D test and F test whereas Tajima’s D test was negative but not statistically significant (Table 2), suggesting population expansion or purifying selection.

Among individual genes, RdRp exhibited the highest nucleotide diversity (π = 0.00764), as well as greater haplotype diversity, high number of haplotypes, mutations, and segregating sites compared to p42. Neutrality tests for p42 mirrored those of the complete genomes, showing significant negative values for Fu and Li’s D and Fu and Li’s F tests and a non-significant negative value for Tajima’s D test. In the case of RdRp, Fu and Li’s D was significantly negative, while Fu and Li’s F and Tajima’s D were not significant. The fusion protein gene had a nucleotide diversity of π = 0.00741, and all neutrality tests returned negative values, with Fu and Li’s D test and Fu and Li’s F test being statistically significant. Interestingly, the results for the fusion protein gene were identical to those obtained for the complete genomes of STV.

For RdRp, K_s_* was 1.68705, K_st_* 0.30733, and Z* 7.16782, indicating clear genetic differentiation between the two phylogroups, which was further supported by a significant S_nn_ value of 1.00000. Similarly, for p42, K_s_* 1.07057, K_st_* 0.33989, and Z* 7.63219 indicated strong differentiation, with S_nn_ 0.99231 confirming the result. The high Fst values (0.81010 for RdRp and 0.80377 for p42) and low migration rates (Nm = 0.06) further support the pronounced genetic divergence between these phylogroups.

### 3.4. Recombination in the STV Population

Recombination detection based on complete genome alignment revealed four potential recombination events. These were identified using RDP v4.101, applying multiple algorithms (MaxChi, SiScan, and 3Seq). The first event involved a single isolate, accession OR725027 (China) with accession PQ492143 (Fiji) and accession KT438549 (China) identified as the major and minor parents, respectively. This event was supported by three different methods (MaxChi, SiScan and 3Seq) and a significant *p*-value of 2.766 × 10^−4^ (3Seq). The second recombination event, supported by two methods (MaxChi and 3Seq), was found in accession OL471984 (Slovenia) with OL471992 (Slovenia) as major parent and PV786595 (OK isolate, this study) as the minor parent (*p* = 1.739 × 10^−3^, 3Seq). A third event, detected by MaxChi alone, involved accession KT438549 (China) with an unknown major parent and PV786595 (OK isolate, this study), as the minor parent. The fourth recombination event was identified by SiScan, Chimaera, and 3Seq, with OK309713 (Turkey) designated as the recombinant, PQ429143 (Fiji) as the minor parent, and OK309721 (Turkey) as major parent (*p* = 4.423 × 10^−2^, Chimaera). The program warned for the 2nd and 4th recombination events that these could be attributed to other evolutionary events. None of these events were considered significant, as they were not detected by at least four different methods.

Additionally, GARD analysis detected a single recombination breakpoint in the complete genome alignment, with model comparison yielding Δc-AIC values of 65.4360 (vs. null model) and 343.678 (vs. the single tree multiple partition model), pinpointing the recombination breakpoint near nucleotide position 1540.

### 3.5. Bayesian Phylogenetic Analysis and Substitution Rate

**Figure 2:**
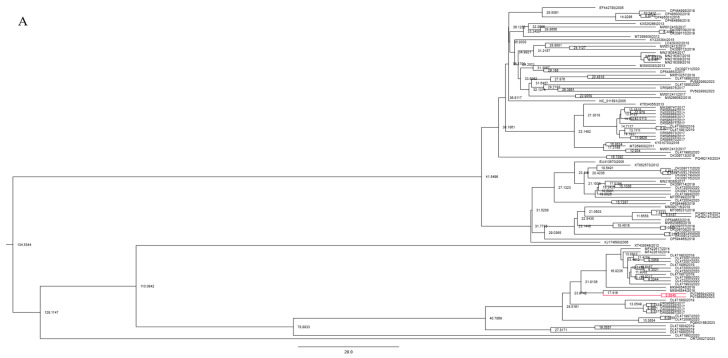

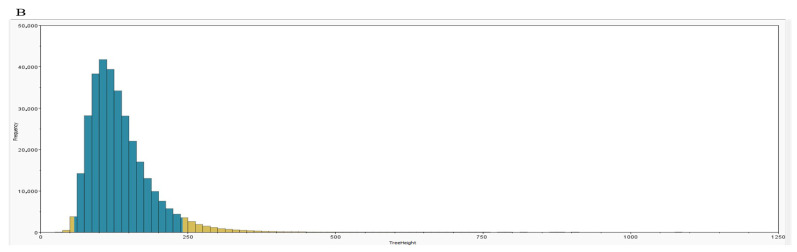
Bayesian based analysis of 104 fusion protein nucleotide sequences of southern tomato virus (STV). (**A**) Time-scaled Bayesian maximum clade credibility tree constructed from fusion protein sequences of STV isolates. (**B**) Posterior distribution of tree height estimated using BEAST analysis of fusion protein gene sequences of STV isolates with blue representing sampling falling within the 95% posterior probability range while yellow is outside that range. (Branches highlighted in red indicate STV isolates obtained in the present study).

Bayesian phylogenetic analyses were conducted to estimate the evolutionary timelines and substitution rate of STV genes. All estimated sample sizes (ESS) exceeded 200, indicating adequate sampling and convergence of parameters. The maximum clade credibility (MCC) tree (Figure 2A) derived from the fusion protein gene (*n* = 104) estimated the time to the most recent common ancestor (TMRCA) at approximately 135 years ago (circa 1889), with 95% highest posterior density (HPD) interval spans from 56.9 to 241.4 years ago (Figure 2B). The mean substitution rate of 6.514 × 10^−5^ substitutions per site per year (Table 3).

Similarly, the RdRp gene exhibited a mean substitution rate of 4.867 × 10^−5^ substitutions per site per year (Table 3), and its TMRCA was estimated at 188.3 years ago (circa 1836) (Figure S9).

In contrast, the p42 gene showed a higher mean substitution rate of 1.184 × 10^−4^ substitutions per site per year (Table 3) and a more recent TMRCA of approximately 58.5 years ago (circa 1965) (Figure S10). The MCC trees for the fusion protein, RdRp (Figure S11), and p42 (Figure S12) genes were largely consistent with their respective ML trees, with a minor exception involving a few isolates.

### 3.6. Comparison of Intrinsically Disordered Regions in STV Proteins

Intrinsically disordered proteins (IDPs) and intrinsically disordered protein regions are known to facilitate protein–protein interactions (PPIs), including complex formation and roles in transcription and translation [45,46]. Analysis of STV proteins using IUPred2A revealed that p42 had the highest proportion of disordered amino acids residues (23.34%) (Figure S13), followed by RdRp (3.81%) (Figure S14) and the fusion protein (2.92%) (Figure S15). Most disordered residues were concentrated at the C-terminal regions of all three proteins. Specifically, 98.86% of disordered residues in p42, 93.10% in RdRp, and 87.10% in the fusion protein were localized to the C-terminal region, whereas only minor fractions (1.14%, 6.90%, and 12.90%, respectively) were observed in the N-terminal regions. Statistical comparison using the Wilcoxon rank-sum test indicated no significant differences (α = 0.05) in the percentage of disordered residues between the study isolates and the reference genome.

### 3.7. Selective Pressure Analysis

For p42, FUBAR identified evidence of pervasive positive (diversifying) selection at four codon sites and pervasive negative (purifying) selection at twenty codon sites, using a posterior probability threshold of 0.9. For RdRp, eight codon sites showed evidence of positive selection, while twenty-two sites were under purifying selection. For fusion protein, thirty-four sites were found to be under purifying selection while four sites were under diversifying selection. These results suggest that although purifying selection is the dominant evolutionary force acting on both genes, a subset of codon positions may be experiencing adaptive evolutionary pressures.

SLAC analysis revealed no evidence of pervasive positive (diversifying) selection in the RdRp or p42 genes at a significance level of 0.1. However, it detected pervasive negative (purifying) selection at sixteen codon sites in the RdRp gene and at six codon sites in the p42 gene. In contrast, the fusion protein gene showed one site under positive selection and twenty-four sites under purifying selection. MEME analysis identified evidence of episodic diversifying selection at a single site in each of the p42, RdRp, and fusion protein genes, based on the likelihood ratio test with a significance threshold of *p* ≤ 0.1.

## 4. Discussion

Viruses are characterized by their high evolutionary rate, enabling rapid mutation and contributing to the extensive genetic diversity observed in viral genomes. These evolutionary imprints can be effectively traced through analyses of viral evolutionary dynamics. In tomato, the diversity of viral pathogens has significantly expanded in recent years, with over 312 viruses so far [47]. STV is among the persistent RNA viruses affecting tomato, transmitted vertically through tomato seeds rather than mechanically or via grafting. Although no confirmed vector has been identified, earlier studies have hypothesized a potential role of insect vectors in STV transmission [6].

Interestingly, STV has been reported to exhibit mutualistic effects in single infected tomato plants, with positive impacts on certain plant growth parameters [48]. However, when co-infected with other viruses, STV is associated with more severe disease symptoms [11,49].

In this study, we performed a comprehensive analysis of STV isolates identified for the first time in Oklahoma, alongside publicly available global isolates. Analyses included assessment of genomic variations, recombination, nucleotide substitution rate, disordered protein regions, and phylogenetic relationships. Phylogenetic trees constructed from the complete genome and individual genes (p42, RdRp, and the fusion protein) consistently revealed two primary clades, independent of host or geographic origin. These findings align with previous studies [11,18,21,25]. However, the Oklahoma isolates clustered within Clade II (based on complete genome and individual genes), in contrast to previously reported U.S. isolates, which were grouped in Clade I. This divergence may reflect the introduction of contaminated tomato seeds through global trade as hypothesized in the case of tomato brown rugose fruit virus (ToBRFV) in Netherlands [50], or it may result from local viral evolution overtime due to the error-prone nature of RdRp which lacks proofreading capabilities [51]. Another possible contributor to genetic divergence may be the adaptation of an unidentified vector, if one is involved in transmission, as speculated previously [6]. The presence of two distinct STV subpopulations within Slovenia isolates, as observed in our study, support the hypothesis of concurrent viral lineages within a single geographic region.

Bayesian coalescent analysis of the fusion protein gene estimated the most recent common ancestor of STV to exist around 135 years ago (circa 1889) with a 95% highest posterior density (HPD) interval ranging from 56.8 to 241.4 years. This timeframe predates the first reported case of ‘‘tomato decline’’ in California’s Imperial Valley in 1984, where virus-like symptoms such as yellowing, decline, and poor fruit set were observed [52]. Given STV’s cryptic nature and its frequent detection in asymptomatic infections [6,11,49,53], it is plausible that the virus remained undetected for decades. STV is frequently detected in mixed infections, particularly with the advent of high-throughput sequencing technologies [8,10,11,12,14,15,16,18,19,21,22,54,55]. STV, in association with other viruses including tomato yellow leaf curl virus (TYLCV), tomato chlorosis virus (ToCV), tomato infectious chlorosis virus (TICV), pepino mosaic virus (PepMV), cucumber mosaic virus (CMV), tomato mosaic virus (ToMV), and tomato spotted wilt virus (TSWV), can cause tomato yellow stunt disease (ToYSD) [6,13,14,17,20,24]. STV interacts with CMV and PepMV in mixed infections, enhancing the pathogenic effects of these viruses [49]. Mixed infections involving unrelated viruses within a host can lead to the emergence of new diseases or enhance the pathogenicity of the co-infecting viruses [56,57]. Since mixed infections are common under natural conditions, the interactions between pathogenic and persistent plant viruses warrant further in-depth study [18].

BEAST analysis based on RdRp and p42 estimated TMRCAs of approximately 188.3 and 58.5 years ago, respectively. The evolutionary history of viruses in the family Amalgaviridae suggests an important role of recombination, with evidence pointing to gene flow between double-stranded RNA viruses (e.g., *Partitiviridae*) and negative-strand RNA viruses (e.g., *Phlebovirus* and *Tenuivirus*) [58]. Further phylogenomic studies, including representative taxa from these families, may offer deeper insight into genomic architecture and inter-viral gene exchanges that have shaped amalgavirus evolution. The estimated substitution rate for the STV RdRp gene was 4.866 × 10^−5^ substitutions per site per year. Research on substitution rates for dsRNA viruses is limited [59]; however, experimental studies on dsRNA bacteriophage φ6 have reported mutation rates in the range of 10^−4^ [60]. These findings suggest that dsRNA viruses may exhibit lower substitutions rates, particularly in plant hosts, where virus evolution tends to proceed more slowly than in animal-infecting viruses [59]. Further comparative research is needed to elucidate evolutionary constraints and dynamics specific to plant dsRNA viruses.

Population structure analysis revealed relatively low genetic diversity among STV isolates. The p42 gene exhibited lower nucleotide diversity (π = 0.00550) than RdRp (π = 0.00764) which also demonstrate greater haplotype diversity and a higher number of segregating sites. These results are consistent with earlier reports [11,18,21,25]. Future studies could investigate how different open reading frames (ORFs) contribute to overall viral diversity. Similar approaches have been employed successfully to study coat protein gene in soybean mosaic virus [61] and the VPg gene in potato potyvirus Y [62].

Selection pressure analysis using FUBAR and SLAC revealed that purifying selection is the predominant evolutionary force acting on STV genes. FUBAR detected limited positive selection (four codons each in the fusion protein and p42), while identifying twenty-one and thirty-five codons under purifying selection in p42 and the fusion protein, respectively. SLAC analysis corroborated these findings, detecting several codons (twenty-five in fusion protein, six in p42) under purifying selection and only one site under positive selection in the fusion protein. MEME analysis further identified one site in the p42 gene and two sites in the fusion protein gene undergoing episodic positive selection, supporting the presence of lineage-specific adaptive evolution. Neutrality tests (Fu and Li’s D test and Fu and Li’s F test) gave significant negative values across the complete genomes and individual genes, suggesting a dominant role of negative selection. Tajima’s D test values were negative but not statistically significant, possibly due to the transient nature of opposing selection forces, which can reduce the test’s power [63]. These findings are in partial agreement with previous studies, while some previous studies [21,25] reported similar patterns for p42 and fusion proteins genes. Our estimates of Tajima’s D differed from those observed previously [11], potentially due to the larger dataset used in this study.

Population genetic differentiation analyses of both RdRp and p42 genes provided strong evidence for substantial divergence between Clade I and Clade II phylogroups. The highly significant values of genetic differentiation tests (K_s_*, K_st_*, and Z*) for both genes (*p* < 0.001) suggest that these groups are genetically distinct. For the RdRp gene, the relatively high values of K_s_* (1.687) and Z* (7.168), along with a maximum S_nn_ value (1.000), indicate complete genetic segregation between the phylogroups. Similarly, the p42 gene showed strong differentiation, as reflected by a high K_st_* (0.340) and Z* (7.632), and an S_nn_ of 0.992. The Fst values exceeding 0.33 for both RdRp and p42 genes suggests that the majority of genetic variation is distributed between phylogroups rather than within them, reinforcing the presence of strong population structure. Additionally, the low effective number of migrants implies minimal gene flow, which appears insufficient to prevent the accumulation of genetic divergence. Such restricted migration and strong differentiation are consistent with long-term evolutionary separation and may reflect ecological or host-driven barriers that limit genetic exchange. The results of this study are consistent with those of an earlier study [21], which also reported significant K_s_*, K_st_*, and Z* values between the two lineages, with Fst exceeding 0.33 and an absolute migration rate below 1.

Recombination is considered as a major driver of genomic variation in plant RNA viruses [64]. No recombination event was identified in the complete STV genomes in this study, consistent with the previous findings [18,21,25]. In contrast, a previous study [11] identified three recombination events among 44 STV isolates, which may reflect differences in the threshold criteria applied during analysis.

Lack of stable tertiary or three-dimensional structure and proper folding characterizes the disordered protein regions in viruses. IDPRs and IDPs play critical roles in various biological functions such as cellular signaling, cell regulation, survival, differentiation, proliferation, and apoptosis [65,66] due to their high plasticity and flexibility [67,68]. In viruses, IDPRs facilitate adaptability, immune evasions and replication regulation [69,70,71]. Our analysis revealed that p42 contained the highest proportion of disordered amino acids (23.34%) compared to RdRp (3.81%) and the fusion protein (2.92%) with disordered residues primarily located in the C-terminal regions. IDRs are known to tolerate high mutation rates, often resulting in functional polymorphism and adaptive potential [37,72]. This is consistent with findings from other RNA viruses, such as Nodamura virus (NoV), where IDRs play a central role in environmental adaptability [69].

## 5. Conclusions

This study presents the first report of STV in Oklahoma and provides a comprehensive analysis of its genetic diversity and evolutionary characteristics. The phylogenetic placement of Oklahoma isolates in Clade II, distinct from other U.S. isolates, suggests potential introduction via international seed trade or local evolutionary divergence. Further, the BEAST analysis estimated the TMRCA of STV to be approximately 135 years ago. Protein disorder predictions indicated high disorder in p42 C-terminal region, suggesting its involvement in host interaction. Among the genes analyzed, RdRp exhibited higher genetic variability than p42, and all genes were found to be evolving predominantly under purifying selection. These findings emphasize the need for ongoing genomic surveillance and highlight the importance of molecular tools in understanding the epidemiology, evolution, and management of persistent plant viruses such as STV.

## Figures and Tables

**Table 1:** Primers used for the diagnosis of southern tomato virus (STV) using reverse transcription polymerase chain reaction (RT-PCR) assays.

Primer Name	Sequence (5′-3′)	Annealing Temperature (°C)	Amplicon Size (bp)
STV 1F	GCGAGAGCGATAAATTTAGTAAGCTAC	53	673
STV 1R	TTGACAATCTTACGCTGCAGATCAG		
STV 2F	GAGAAGAGGACACTGCAGTACAA	54	503
STV 2R	GTAGATATCCTCCATCAGACTCT		

**Table 2:** Analysis of genetic diversity within the complete genomes and individual genes of southern tomato virus (STV) isolates.

Gene	No. of Sequences	Total no. of Nucleotide Sites	No. of Nucleotide Sites ^a^	S	H	H_d_	k	π	Eta	Theta (Per Sequence)	Theta (Per Site)	Tajima’s Test	Fu and Li’s D Test	Fu and Li’s F Test
Complete genome	108	3475	3190	184	70	0.979	23.64123	0.00741	193	36.72895	0.01151	−1.18568 ^NS^	−3.22915 *	−2.79709 *
p42	130	1134	1134	67	47	0.898	6.23280	0.00550	69	12.68173	0.01118	−1.60530 ^NS^	−3.21261 *	−3.02698 *
RdRp	108	2289	2289	129	60	0.960	17.48165	0.00764	138	26.26215	0.01147	−1.10373 ^NS^	−2.62916 *	−2.35996 ^NS^
Fusion protein	108	3190	3190	184	70	0.979	23.64123	0.00741	193	37.72895	0.01151	−1.18568 ^NS^	−3.22915 *	−2.79709 *

^a^ Number of nucleotide sites excluding gaps and missing data. k, Average number of nucleotide differences. S, number of segregating (polymorphic) sites. H, number of haplotypes. Eta, the total number of mutations. H_d_, haplotype diversity. π, nucleotide diversity. *, statistically significant (*p* ≤ 0.05). NS, Statistically non-significant (0.10 > *p* > 0.05).

**Table 3:** Estimates of nucleotide substitution rate and the age of diversity for southern tomato virus (STV) isolates.

Gene	Date Range	Mean Substitution Rate (Subs/Site/Year)	HPD ^a^	TMRCA ^b^	HPD ^a^
Fusion protein	2005–2024	6.514 × 10^−5^	3.4575 × 10^−5^–9.6661 × 10^−5^	135.137	56.8965–241.3992
p42	2005–2024	1.184 × 10^−4^	5.7164 × 10^−5^–1.8674 × 10^−4^	58.546	23.4486–102.1011
RdRp	2005–2024	4.866 × 10^−5^	2.0639 × 10^−5^–7.7596 × 10^−5^	188.263	64.1967–368.4334

^a^ 95% Highest Probability Density (HPD) values. ^b^ Time to the Most Common Ancestor (TMRCA); years ago.

## Data Availability

The STV genomes obtained in this study have been submitted to GenBank under accession numbers PV786594 (OK1) and PV786595 (OK2) and are publicly available at https://www.ncbi.nlm.nih.gov/genbank (accessed on 29 June 2025).

## Supplementary Materials

The following supporting information can be downloaded at: https://www.mdpi.com/article/10.3390/v17091193/s1, **Figure S1**: Agarose gel electrophoresis of PCR products amplified using the STV1 primer pair. Lane 1: molecular weight marker (100 bp Plus); lanes 2–6: tomato samples K71–K75; lane 7: healthy tomato control; lane 8: negative control (water); **Figure S2**: Agarose gel electrophoresis of PCR products amplified using the STV2 primer pair. Lane 1: molecular weight marker (100 bp Plus); lanes 2–6: tomato samples K71–K75; lane 7: healthy tomato control; lane 8: negative control (water); **Figure S3**: Phylogenetic tree of 108 STV fusion protein gene nucleotide sequences constructed using maximum likelihood with 1,000 bootstrap replicates; **Figure S4**: Maximum likelihood phylogenetic tree with 1000 bootstrap replicates using 108 nucleotide sequences of RdRp gene of STV; **Figure S5**: Maximum likelihood phylogenetic tree of 130 STV p42 gene nucleotide sequences with 1000 bootstrap replicates; **Figure S6**: Nucleotide pairwise identity heatmap of fusion protein gene sequences. (Highlighted in red color or asterisk-marked (*) STV isolates represent those identified in this study); **Figure S7**: Nucleotide pairwise identity heatmap of RdRp gene sequences. (Highlighted in red color or asterisk-marked (*) STV isolates represent those identified in this study); **Figure S8**: Nucleotide pairwise identity heatmap of p42 gene sequences. (Highlighted in red color or asterisk-marked (*) STV isolates represent those identified in this study); **Figure S9**: Posterior distribution of tree height inferred from BEAST analysis of 104 RdRp gene sequences of STV isolates; **Figure S10**: Posterior distribution of tree height estimated using BEAST analysis of 110 p42 gene sequences of STV isolates; **Figure S11**: Time-scaled Bayesian maximum clade credibility tree of RdRp sequences of STV. (Branches highlighted in red indicate STV isolates obtained in the present study); **Figure S12**: Time-scaled Bayesian maximum clade credibility tree based on p42 sequences of STV. (Branches highlighted in red indicate STV isolates obtained in the present study); **Figure S13**: Distribution of ordered and disordered amino acid residues within p42 protein of the southern tomato virus (STV) isolates. The STV isolates analyzed were (A) PV786594-OK1, (B) PV786595-OK2, and (C) NC_011591; **Figure S14**: Distribution of ordered and disordered amino acid residues in RdRp protein of southern tomato virus (STV) isolates. The analyzed isolates include (A) PV786594-OK1, (B) PV786595-OK2, and (C) NC_011591. **Figure S15**: Distribution of ordered and disordered amino acid residues across fusion protein of southern tomato virus (STV) isolates. The STV isolates analyzed were (A) PV786594-OK1, (B) PV786595-OK2, and (C) NC_011591.
