# Supplementary material for: First Detection and Identification of Southern Tomato Virus Infecting Tomatoes in Oklahoma with Complete Genome Characterization and Insights into Global Genetic Diversity
Source: Viruses. 2025 Aug 30;17(9):1193. doi: 10.3390/v17091193 (PMC12474347; doi:10.3390/v17091193)
Supplement: Supplementary file 1 [file viruses-17-01193-s001.zip › viruses-3786042-supplementary.pptx]

## Slide 1
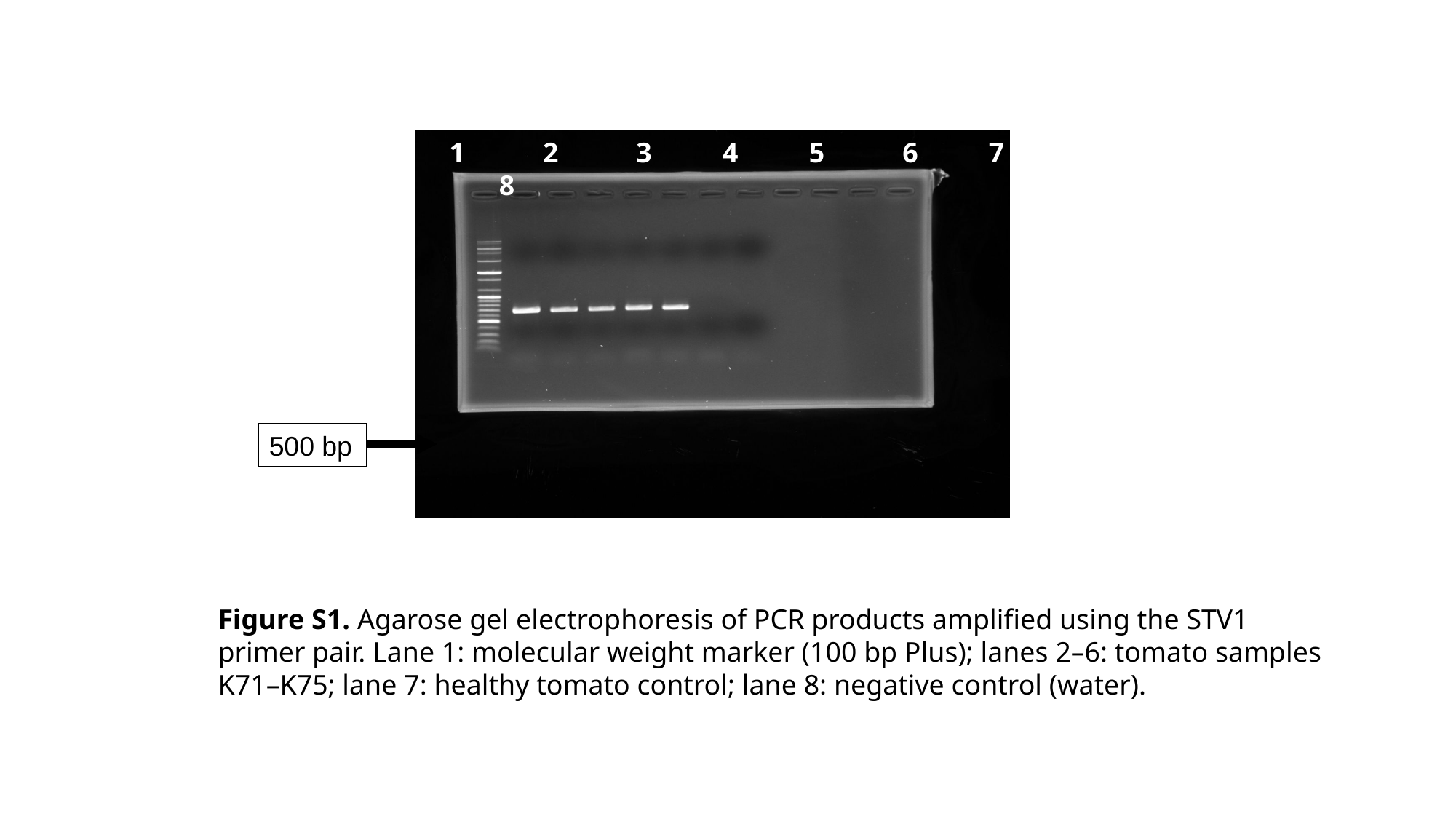

1 2 3 4 5 6 7 8
500 bp
Figure S1. Agarose gel electrophoresis of PCR products amplified using the STV1 primer pair. Lane 1: molecular weight marker (100 bp Plus); lanes 2–6: tomato samples K71–K75; lane 7: healthy tomato control; lane 8: negative control (water).

## Slide 2
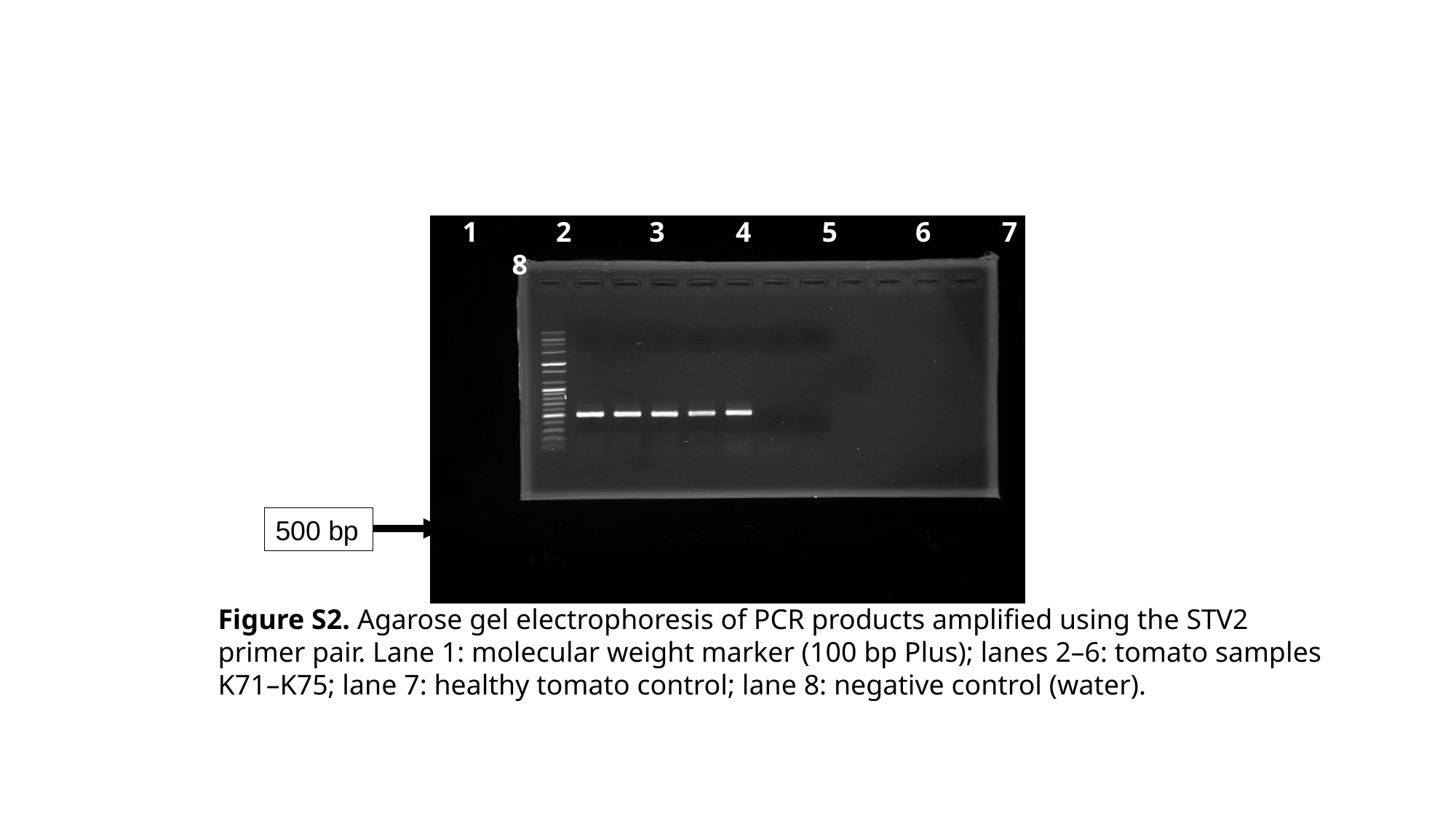

1 2 3 4 5 6 7 8
500 bp
Figure S2. Agarose gel electrophoresis of PCR products amplified using the STV2 primer pair. Lane 1: molecular weight marker (100 bp Plus); lanes 2–6: tomato samples K71–K75; lane 7: healthy tomato control; lane 8: negative control (water).

## Slide 3
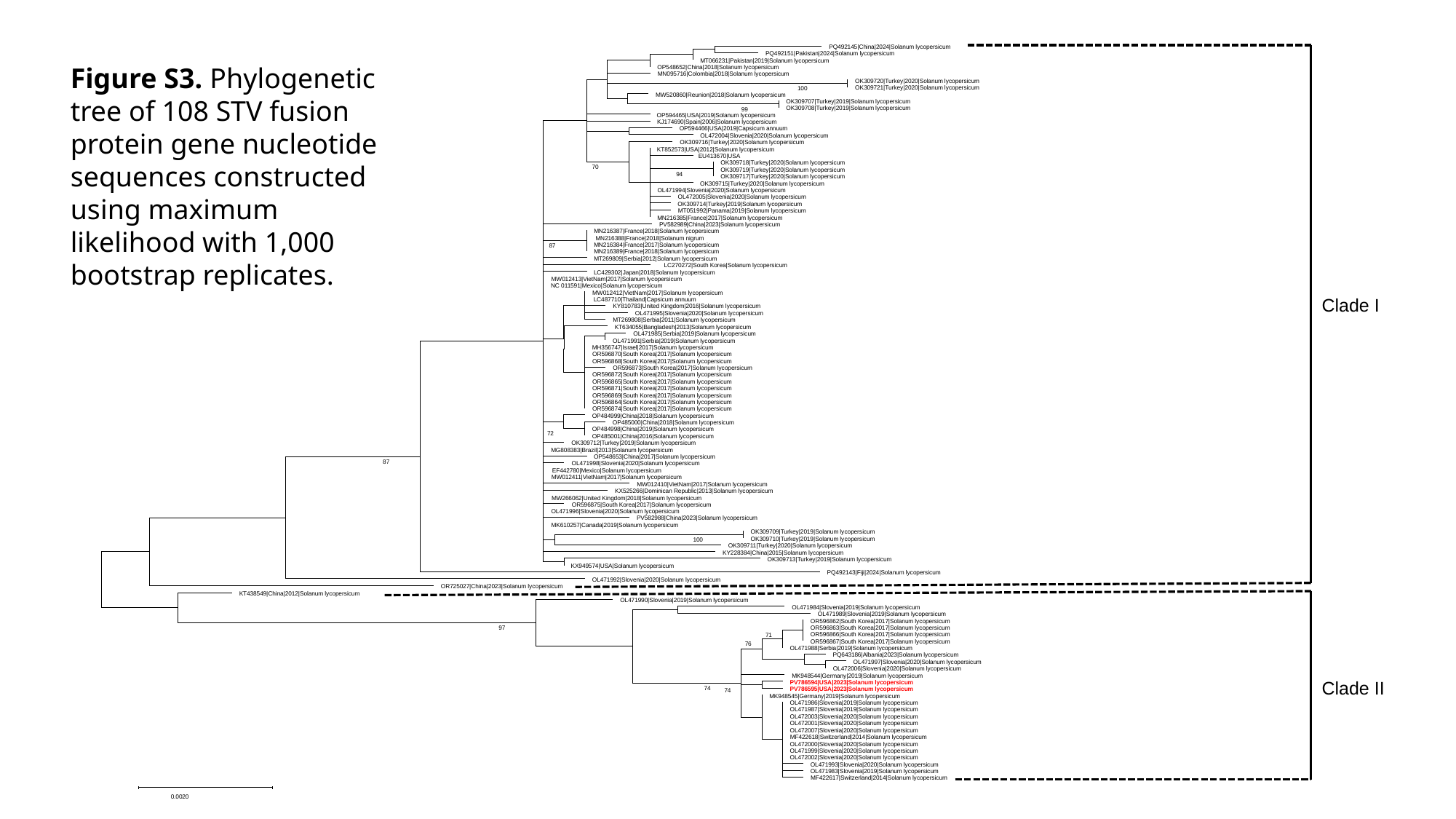

PQ492145|China|2024|Solanum lycopersicum
 PQ492151|Pakistan|2024|Solanum lycopersicum
 MT066231|Pakistan|2019|Solanum lycopersicum
 OP548652|China|2018|Solanum lycopersicum
 MN095716|Colombia|2018|Solanum lycopersicum
 OK309720|Turkey|2020|Solanum lycopersicum
 OK309721|Turkey|2020|Solanum lycopersicum
 MW520860|Reunion|2018|Solanum lycopersicum
 OK309707|Turkey|2019|Solanum lycopersicum
 OK309708|Turkey|2019|Solanum lycopersicum
 OP594465|USA|2019|Solanum lycopersicum
 KJ174690|Spain|2006|Solanum lycopersicum
 OP594466|USA|2019|Capsicum annuum
 OL472004|Slovenia|2020|Solanum lycopersicum
 OK309716|Turkey|2020|Solanum lycopersicum
 KT852573|USA|2012|Solanum lycopersicum
 EU413670|USA
 OK309718|Turkey|2020|Solanum lycopersicum
 OK309719|Turkey|2020|Solanum lycopersicum
 OK309717|Turkey|2020|Solanum lycopersicum
 OK309715|Turkey|2020|Solanum lycopersicum
 OL471994|Slovenia|2020|Solanum lycopersicum
 OL472005|Slovenia|2020|Solanum lycopersicum
 OK309714|Turkey|2019|Solanum lycopersicum
 MT051992|Panama|2019|Solanum lycopersicum
 MN216385|France|2017|Solanum lycopersicum
 PV582989|China|2023|Solanum lycopersicum
 MN216387|France|2018|Solanum lycopersicum
 MN216388|France|2018|Solanum nigrum
 MN216384|France|2017|Solanum lycopersicum
 MN216389|France|2018|Solanum lycopersicum
 MT269809|Serbia|2012|Solanum lycopersicum
 LC270272|South Korea|Solanum lycopersicum
 LC429302|Japan|2018|Solanum lycopersicum
 MW012413|VietNam|2017|Solanum lycopersicum
 NC 011591|Mexico|Solanum lycopersicum
 MW012412|VietNam|2017|Solanum lycopersicum
 LC487710|Thailand|Capsicum annuum
 KY810783|United Kingdom|2016|Solanum lycopersicum
 OL471995|Slovenia|2020|Solanum lycopersicum
 MT269808|Serbia|2011|Solanum lycopersicum
 KT634055|Bangladesh|2013|Solanum lycopersicum
 OL471985|Serbia|2019|Solanum lycopersicum
 OL471991|Serbia|2019|Solanum lycopersicum
 MH356747|Israel|2017|Solanum lycopersicum
 OR596870|South Korea|2017|Solanum lycopersicum
 OR596868|South Korea|2017|Solanum lycopersicum
 OR596873|South Korea|2017|Solanum lycopersicum
 OR596872|South Korea|2017|Solanum lycopersicum
 OR596865|South Korea|2017|Solanum lycopersicum
 OR596871|South Korea|2017|Solanum lycopersicum
 OR596869|South Korea|2017|Solanum lycopersicum
 OR596864|South Korea|2017|Solanum lycopersicum
 OR596874|South Korea|2017|Solanum lycopersicum
 OP484999|China|2018|Solanum lycopersicum
 OP485000|China|2018|Solanum lycopersicum
 OP484998|China|2019|Solanum lycopersicum
 OP485001|China|2016|Solanum lycopersicum
 OK309712|Turkey|2019|Solanum lycopersicum
 MG808383|Brazil|2013|Solanum lycopersicum
 OP548653|China|2017|Solanum lycopersicum
 OL471998|Slovenia|2020|Solanum lycopersicum
 EF442780|Mexico|Solanum lycopersicum
 MW012411|VietNam|2017|Solanum lycopersicum
 MW012410|VietNam|2017|Solanum lycopersicum
 KX525266|Dominican Republic|2013|Solanum lycopersicum
 MW266062|United Kingdom|2018|Solanum lycopersicum
 OR596875|South Korea|2017|Solanum lycopersicum
 OL471996|Slovenia|2020|Solanum lycopersicum
 PV582988|China|2023|Solanum lycopersicum
100
99
70
94
87
72
87
 MK610257|Canada|2019|Solanum lycopersicum
 OK309709|Turkey|2019|Solanum lycopersicum
 OK309710|Turkey|2019|Solanum lycopersicum
100
 OK309711|Turkey|2020|Solanum lycopersicum
 KY228384|China|2015|Solanum lycopersicum
 OK309713|Turkey|2019|Solanum lycopersicum
 KX949574|USA|Solanum lycopersicum
 PQ492143|Fiji|2024|Solanum lycopersicum
 OL471992|Slovenia|2020|Solanum lycopersicum
 OR725027|China|2023|Solanum lycopersicum
 KT438549|China|2012|Solanum lycopersicum
 OL471990|Slovenia|2019|Solanum lycopersicum
 OL471984|Slovenia|2019|Solanum lycopersicum
 OL471989|Slovenia|2019|Solanum lycopersicum
 OR596862|South Korea|2017|Solanum lycopersicum
 OR596863|South Korea|2017|Solanum lycopersicum
97
 OR596866|South Korea|2017|Solanum lycopersicum
71
 OR596867|South Korea|2017|Solanum lycopersicum
76
 OL471988|Serbia|2019|Solanum lycopersicum
 PQ643186|Albania|2023|Solanum lycopersicum
 OL471997|Slovenia|2020|Solanum lycopersicum
 OL472006|Slovenia|2020|Solanum lycopersicum
 MK948544|Germany|2019|Solanum lycopersicum
 PV786594|USA|2023|Solanum lycopersicum
74
 PV786595|USA|2023|Solanum lycopersicum
74
 MK948545|Germany|2019|Solanum lycopersicum
 OL471986|Slovenia|2019|Solanum lycopersicum
 OL471987|Slovenia|2019|Solanum lycopersicum
 OL472003|Slovenia|2020|Solanum lycopersicum
 OL472001|Slovenia|2020|Solanum lycopersicum
 OL472007|Slovenia|2020|Solanum lycopersicum
 MF422618|Switzerland|2014|Solanum lycopersicum
 OL472000|Slovenia|2020|Solanum lycopersicum
 OL471999|Slovenia|2020|Solanum lycopersicum
 OL472002|Slovenia|2020|Solanum lycopersicum
 OL471993|Slovenia|2020|Solanum lycopersicum
 OL471983|Slovenia|2019|Solanum lycopersicum
 MF422617|Switzerland|2014|Solanum lycopersicum
0.0020
Clade I
Clade II
Figure S3. Phylogenetic tree of 108 STV fusion protein gene nucleotide sequences constructed using maximum likelihood with 1,000 bootstrap replicates.

## Slide 4
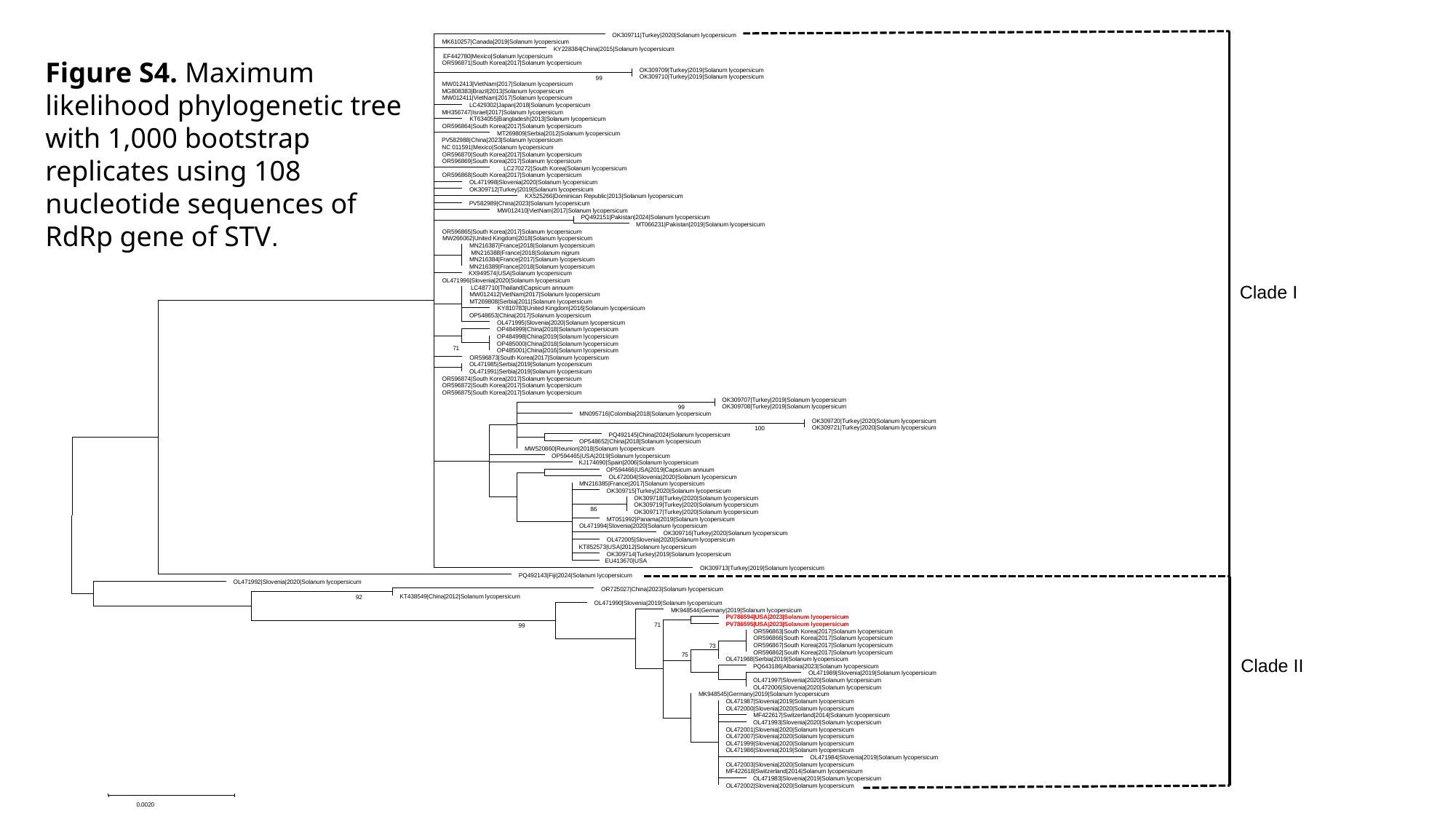

OK309711|Turkey|2020|Solanum lycopersicum
 MK610257|Canada|2019|Solanum lycopersicum
 KY228384|China|2015|Solanum lycopersicum
 EF442780|Mexico|Solanum lycopersicum
 OR596871|South Korea|2017|Solanum lycopersicum
 OK309709|Turkey|2019|Solanum lycopersicum
 OK309710|Turkey|2019|Solanum lycopersicum
 MW012413|VietNam|2017|Solanum lycopersicum
 MG808383|Brazil|2013|Solanum lycopersicum
 MW012411|VietNam|2017|Solanum lycopersicum
 LC429302|Japan|2018|Solanum lycopersicum
 MH356747|Israel|2017|Solanum lycopersicum
 KT634055|Bangladesh|2013|Solanum lycopersicum
 OR596864|South Korea|2017|Solanum lycopersicum
 MT269809|Serbia|2012|Solanum lycopersicum
 PV582988|China|2023|Solanum lycopersicum
 NC 011591|Mexico|Solanum lycopersicum
 OR596870|South Korea|2017|Solanum lycopersicum
 OR596869|South Korea|2017|Solanum lycopersicum
 LC270272|South Korea|Solanum lycopersicum
 OR596868|South Korea|2017|Solanum lycopersicum
 OL471998|Slovenia|2020|Solanum lycopersicum
 OK309712|Turkey|2019|Solanum lycopersicum
 KX525266|Dominican Republic|2013|Solanum lycopersicum
 PV582989|China|2023|Solanum lycopersicum
 MW012410|VietNam|2017|Solanum lycopersicum
 PQ492151|Pakistan|2024|Solanum lycopersicum
 MT066231|Pakistan|2019|Solanum lycopersicum
 OR596865|South Korea|2017|Solanum lycopersicum
 MW266062|United Kingdom|2018|Solanum lycopersicum
 MN216387|France|2018|Solanum lycopersicum
 MN216388|France|2018|Solanum nigrum
 MN216384|France|2017|Solanum lycopersicum
 MN216389|France|2018|Solanum lycopersicum
 KX949574|USA|Solanum lycopersicum
 OL471996|Slovenia|2020|Solanum lycopersicum
 LC487710|Thailand|Capsicum annuum
 MW012412|VietNam|2017|Solanum lycopersicum
 MT269808|Serbia|2011|Solanum lycopersicum
 KY810783|United Kingdom|2016|Solanum lycopersicum
 OP548653|China|2017|Solanum lycopersicum
 OL471995|Slovenia|2020|Solanum lycopersicum
 OP484999|China|2018|Solanum lycopersicum
 OP484998|China|2019|Solanum lycopersicum
 OP485000|China|2018|Solanum lycopersicum
 OP485001|China|2016|Solanum lycopersicum
 OR596873|South Korea|2017|Solanum lycopersicum
 OL471985|Serbia|2019|Solanum lycopersicum
 OL471991|Serbia|2019|Solanum lycopersicum
 OR596874|South Korea|2017|Solanum lycopersicum
 OR596872|South Korea|2017|Solanum lycopersicum
 OR596875|South Korea|2017|Solanum lycopersicum
 OK309707|Turkey|2019|Solanum lycopersicum
 OK309708|Turkey|2019|Solanum lycopersicum
 MN095716|Colombia|2018|Solanum lycopersicum
 OK309720|Turkey|2020|Solanum lycopersicum
 OK309721|Turkey|2020|Solanum lycopersicum
 PQ492145|China|2024|Solanum lycopersicum
 OP548652|China|2018|Solanum lycopersicum
 MW520860|Reunion|2018|Solanum lycopersicum
 OP594465|USA|2019|Solanum lycopersicum
 KJ174690|Spain|2006|Solanum lycopersicum
 OP594466|USA|2019|Capsicum annuum
 OL472004|Slovenia|2020|Solanum lycopersicum
 MN216385|France|2017|Solanum lycopersicum
 OK309715|Turkey|2020|Solanum lycopersicum
 OK309718|Turkey|2020|Solanum lycopersicum
 OK309719|Turkey|2020|Solanum lycopersicum
 OK309717|Turkey|2020|Solanum lycopersicum
 MT051992|Panama|2019|Solanum lycopersicum
 OL471994|Slovenia|2020|Solanum lycopersicum
 OK309716|Turkey|2020|Solanum lycopersicum
 OL472005|Slovenia|2020|Solanum lycopersicum
 KT852573|USA|2012|Solanum lycopersicum
 OK309714|Turkey|2019|Solanum lycopersicum
 EU413670|USA
 OK309713|Turkey|2019|Solanum lycopersicum
 PQ492143|Fiji|2024|Solanum lycopersicum
 OL471992|Slovenia|2020|Solanum lycopersicum
 OR725027|China|2023|Solanum lycopersicum
 KT438549|China|2012|Solanum lycopersicum
 OL471990|Slovenia|2019|Solanum lycopersicum
 MK948544|Germany|2019|Solanum lycopersicum
 PV786594|USA|2023|Solanum lycopersicum
 PV786595|USA|2023|Solanum lycopersicum
 OR596863|South Korea|2017|Solanum lycopersicum
99
71
99
100
86
92
71
99
 OR596866|South Korea|2017|Solanum lycopersicum
 OR596867|South Korea|2017|Solanum lycopersicum
73
 OR596862|South Korea|2017|Solanum lycopersicum
75
 OL471988|Serbia|2019|Solanum lycopersicum
 PQ643186|Albania|2023|Solanum lycopersicum
 OL471989|Slovenia|2019|Solanum lycopersicum
 OL471997|Slovenia|2020|Solanum lycopersicum
 OL472006|Slovenia|2020|Solanum lycopersicum
 MK948545|Germany|2019|Solanum lycopersicum
 OL471987|Slovenia|2019|Solanum lycopersicum
 OL472000|Slovenia|2020|Solanum lycopersicum
 MF422617|Switzerland|2014|Solanum lycopersicum
 OL471993|Slovenia|2020|Solanum lycopersicum
 OL472001|Slovenia|2020|Solanum lycopersicum
 OL472007|Slovenia|2020|Solanum lycopersicum
 OL471999|Slovenia|2020|Solanum lycopersicum
 OL471986|Slovenia|2019|Solanum lycopersicum
 OL471984|Slovenia|2019|Solanum lycopersicum
 OL472003|Slovenia|2020|Solanum lycopersicum
 MF422618|Switzerland|2014|Solanum lycopersicum
 OL471983|Slovenia|2019|Solanum lycopersicum
 OL472002|Slovenia|2020|Solanum lycopersicum
0.0020
Clade I
Clade II
Figure S4. Maximum likelihood phylogenetic tree with 1,000 bootstrap replicates using 108 nucleotide sequences of RdRp gene of STV.

## Slide 5
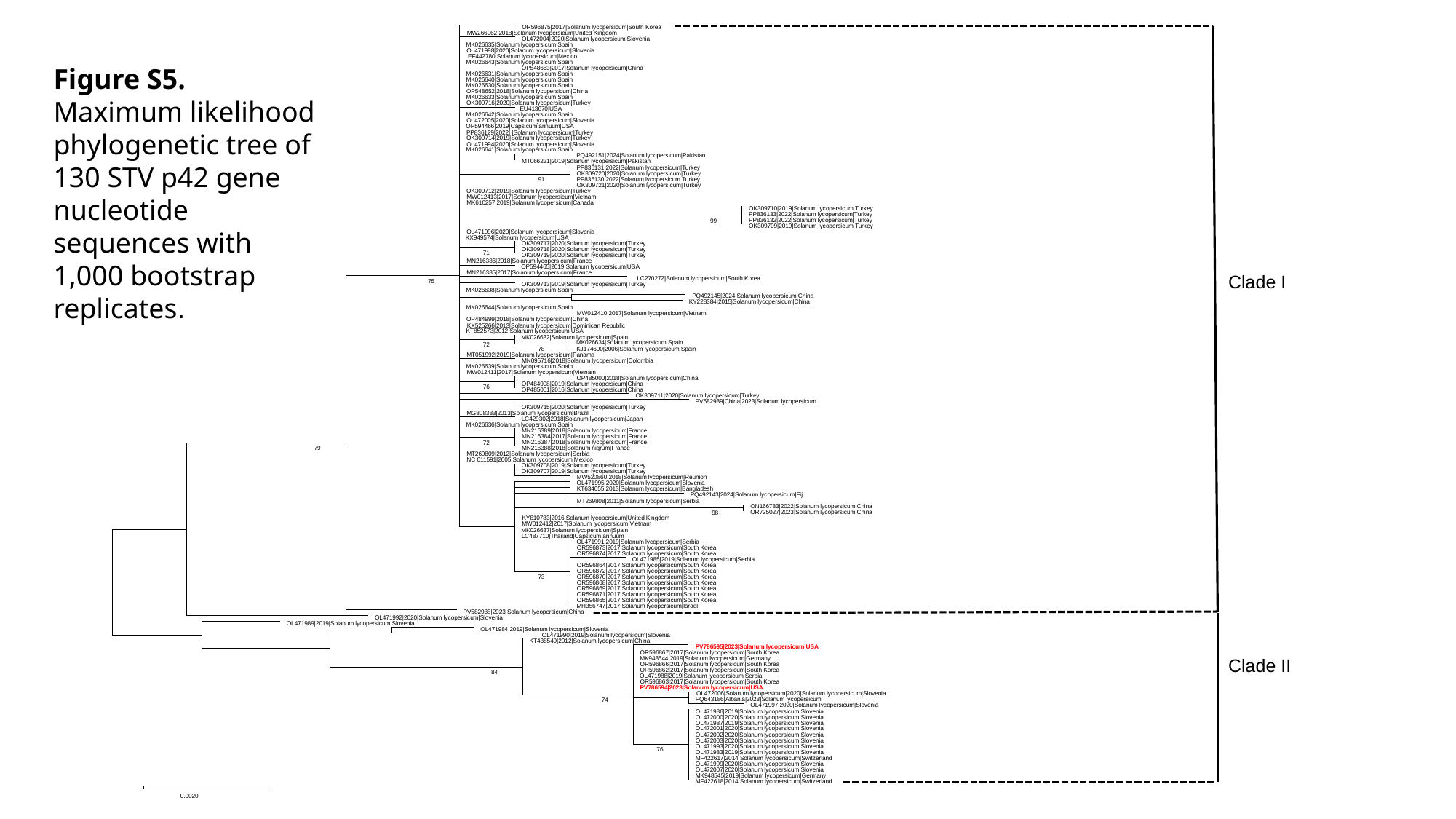

OR596875|2017|Solanum lycopersicum|South Korea
 MW266062|2018|Solanum lycopersicum|United Kingdom
 OL472004|2020|Solanum lycopersicum|Slovenia
 MK026635|Solanum lycopersicum|Spain
 OL471998|2020|Solanum lycopersicum|Slovenia
 EF442780|Solanum lycopersicum|Mexico
 MK026643|Solanum lycopersicum|Spain
 OP548653|2017|Solanum lycopersicum|China
 MK026631|Solanum lycopersicum|Spain
 MK026640|Solanum lycopersicum|Spain
 MK026630|Solanum lycopersicum|Spain
 OP548652|2018|Solanum lycopersicum|China
 MK026633|Solanum lycopersicum|Spain
 OK309716|2020|Solanum lycopersicum|Turkey
 EU413670|USA
 MK026642|Solanum lycopersicum|Spain
 OL472005|2020|Solanum lycopersicum|Slovenia
 OP594466|2019|Capsicum annuum|USA
 PP836129|2022| |Solanum lycopersicum|Turkey
 OK309714|2019|Solanum lycopersicum|Turkey
 OL471994|2020|Solanum lycopersicum|Slovenia
 MK026641|Solanum lycopersicum|Spain
 PQ492151|2024|Solanum lycopersicum|Pakistan
 MT066231|2019|Solanum lycopersicum|Pakistan
 PP836131|2022|Solanum lycopersicum|Turkey
 OK309720|2020|Solanum lycopersicum|Turkey
 PP836130|2022|Solanum lycopersicum Turkey
 OK309721|2020|Solanum lycopersicum|Turkey
 OK309712|2019|Solanum lycopersicum|Turkey
 MW012413|2017|Solanum lycopersicum|Vietnam
 MK610257|2019|Solanum lycopersicum|Canada
 OK309710|2019|Solanum lycopersicum|Turkey
 PP836133|2022|Solanum lycopersicum|Turkey
 PP836132|2022|Solanum lycopersicum|Turkey
 OK309709|2019|Solanum lycopersicum|Turkey
 OL471996|2020|Solanum lycopersicum|Slovenia
 KX949574|Solanum lycopersicum|USA
 OK309717|2020|Solanum lycopersicum|Turkey
 OK309718|2020|Solanum lycopersicum|Turkey
 OK309719|2020|Solanum lycopersicum|Turkey
 MN216386|2018|Solanum lycopersicum|France
 OP594465|2019|Solanum lycopersicum|USA
 MN216385|2017|Solanum lycopersicum|France
 LC270272|Solanum lycopersicum|South Korea
 OK309713|2019|Solanum lycopersicum|Turkey
 MK026638|Solanum lycopersicum|Spain
 PQ492145|2024|Solanum lycopersicum|China
 KY228384|2015|Solanum lycopersicum|China
 MK026644|Solanum lycopersicum|Spain
 MW012410|2017|Solanum lycopersicum|Vietnam
 OP484999|2018|Solanum lycopersicum|China
 KX525266|2013|Solanum lycopersicum|Dominican Republic
 KT852573|2012|Solanum lycopersicum|USA
 MK026632|Solanum lycopersicum|Spain
 MK026634|Solanum lycopersicum|Spain
 KJ174690|2006|Solanum lycopersicum|Spain
 MT051992|2019|Solanum lycopersicum|Panama
 MN095716|2018|Solanum lycopersicum|Colombia
 MK026639|Solanum lycopersicum|Spain
 MW012411|2017|Solanum lycopersicum|Vietnam
 OP485000|2018|Solanum lycopersicum|China
 OP484998|2019|Solanum lycopersicum|China
 OP485001|2016|Solanum lycopersicum|China
 OK309711|2020|Solanum lycopersicum|Turkey
 PV582989|China|2023|Solanum lycopersicum
 OK309715|2020|Solanum lycopersicum|Turkey
 MG808383|2013|Solanum lycopersicum|Brazil
91
99
71
72
78
76
 LC429302|2018|Solanum lycopersicum|Japan
 MK026636|Solanum lycopersicum|Spain
 MN216389|2018|Solanum lycopersicum|France
 MN216384|2017|Solanum lycopersicum|France
 MN216387|2018|Solanum lycopersicum|France
72
 MN216388|2018|Solanum nigrum|France
 MT269809|2012|Solanum lycopersicum|Serbia
 NC 011591|2005|Solanum lycopersicum|Mexico
 OK309708|2019|Solanum lycopersicum|Turkey
 OK309707|2019|Solanum lycopersicum|Turkey
 MW520860|2018|Solanum lycopersicum|Reunion
 OL471995|2020|Solanum lycopersicum|Slovenia
 KT634055|2013|Solanum lycopersicum|Bangladesh
 PQ492143|2024|Solanum lycopersicum|Fiji
 MT269808|2011|Solanum lycopersicum|Serbia
 ON166783|2022|Solanum lycopersicum|China
 OR725027|2023|Solanum lycopersicum|China
98
 KY810783|2016|Solanum lycopersicum|United Kingdom
 MW012412|2017|Solanum lycopersicum|Vietnam
 MK026637|Solanum lycopersicum|Spain
 LC487710|Thailand|Capsicum annuum
 OL471991|2019|Solanum lycopersicum|Serbia
 OR596873|2017|Solanum lycopersicum|South Korea
 OR596874|2017|Solanum lycopersicum|South Korea
 OL471985|2019|Solanum lycopersicum|Serbia
 OR596864|2017|Solanum lycopersicum|South Korea
 OR596872|2017|Solanum lycopersicum|South Korea
 OR596870|2017|Solanum lycopersicum|South Korea
 OR596868|2017|Solanum lycopersicum|South Korea
 OR596869|2017|Solanum lycopersicum|South Korea
 OR596871|2017|Solanum lycopersicum|South Korea
 OR596865|2017|Solanum lycopersicum|South Korea
 MH356747|2017|Solanum lycopersicum|Israel
 PV582988|2023|Solanum lycopersicum|China
 OL471992|2020|Solanum lycopersicum|Slovenia
 OL471989|2019|Solanum lycopersicum|Slovenia
 OL471984|2019|Solanum lycopersicum|Slovenia
 OL471990|2019|Solanum lycopersicum|Slovenia
 KT438549|2012|Solanum lycopersicum|China
 PV786595|2023|Solanum lycopersicum|USA
 OR596867|2017|Solanum lycopersicum|South Korea
 MK948544|2019|Solanum lycopersicum|Germany
 OR596866|2017|Solanum lycopersicum|South Korea
 OR596862|2017|Solanum lycopersicum|South Korea
84
 OL471988|2019|Solanum lycopersicum|Serbia
 OR596863|2017|Solanum lycopersicum|South Korea
 PV786594|2023|Solanum lycopersicum|USA
 OL472006|Solanum lycopersicum|2020|Solanum lycopersicum|Slovenia
 PQ643186|Albania|2023|Solanum lycopersicum
74
 OL471997|2020|Solanum lycopersicum|Slovenia
 OL471986|2019|Solanum lycopersicum|Slovenia
 OL472000|2020|Solanum lycopersicum|Slovenia
 OL471987|2019|Solanum lycopersicum|Slovenia
 OL472001|2020|Solanum lycopersicum|Slovenia
 OL472002|2020|Solanum lycopersicum|Slovenia
 OL472003|2020|Solanum lycopersicum|Slovenia
 OL471993|2020|Solanum lycopersicum|Slovenia
76
 OL471983|2019|Solanum lycopersicum|Slovenia
 MF422617|2014|Solanum lycopersicum|Switzerland
 OL471999|2020|Solanum lycopersicum|Slovenia
 OL472007|2020|Solanum lycopersicum|Slovenia
 MK948545|2019|Solanum lycopersicum|Germany
 MF422618|2014|Solanum lycopersicum|Switzerland
75
79
73
0.0020
Clade I
Clade II
Figure S5. Maximum likelihood phylogenetic tree of 130 STV p42 gene nucleotide sequences with 1,000 bootstrap replicates.

## Slide 6
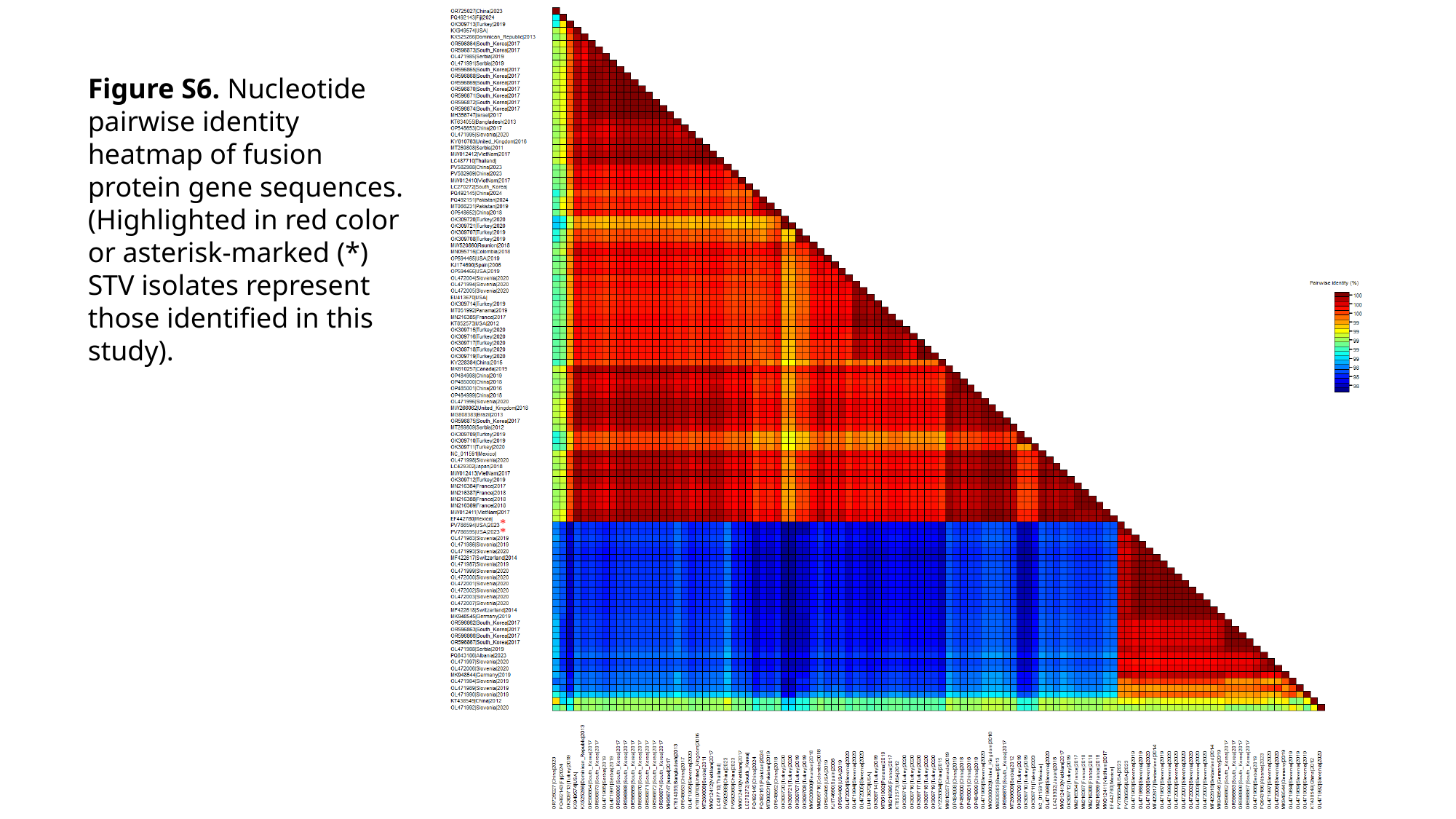

*
*
Figure S6. Nucleotide pairwise identity heatmap of fusion protein gene sequences. (Highlighted in red color or asterisk-marked (*) STV isolates represent those identified in this study).

## Slide 7
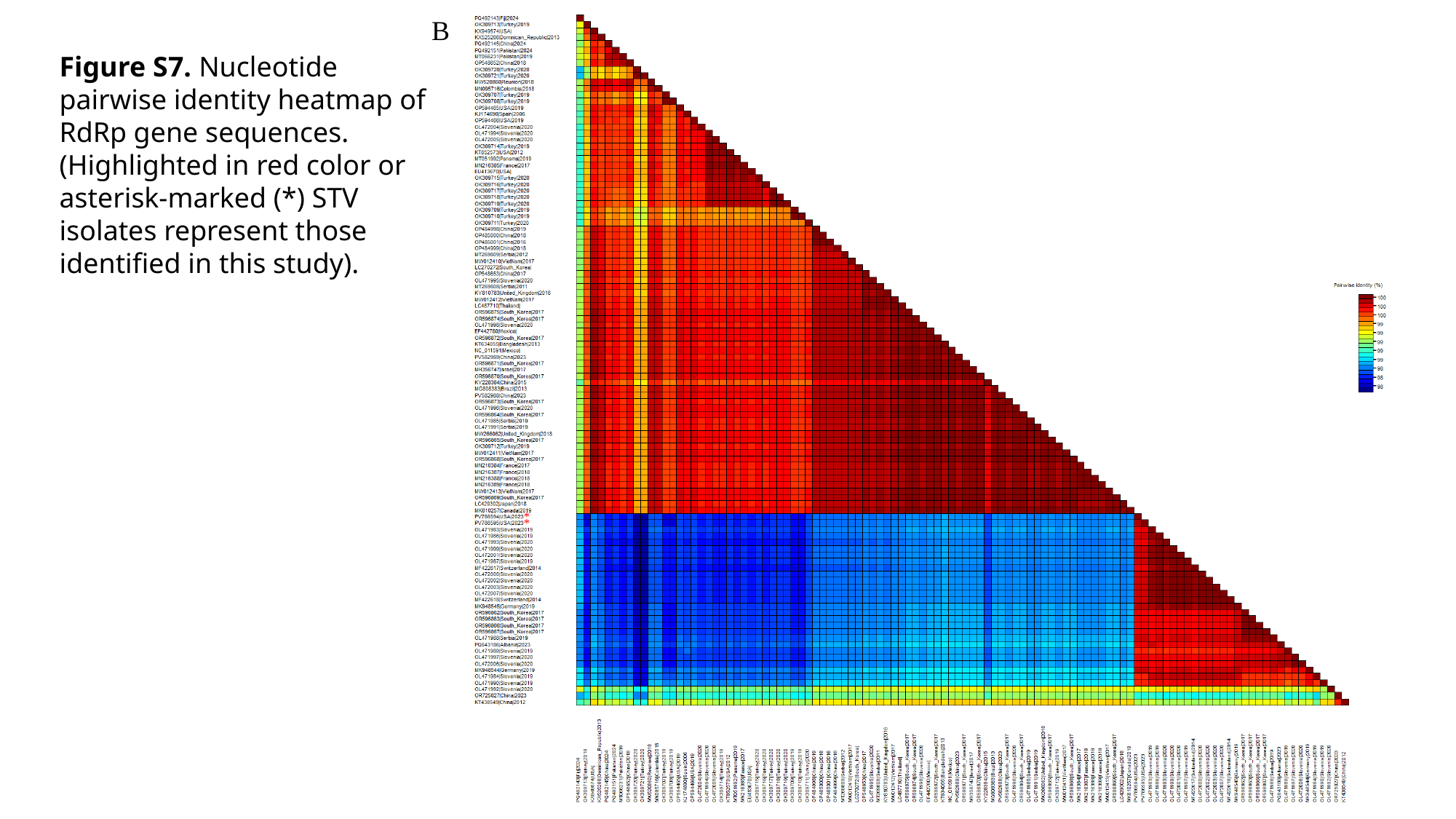

B
*
*
Figure S7. Nucleotide pairwise identity heatmap of RdRp gene sequences. (Highlighted in red color or asterisk-marked (*) STV isolates represent those identified in this study).

## Slide 8
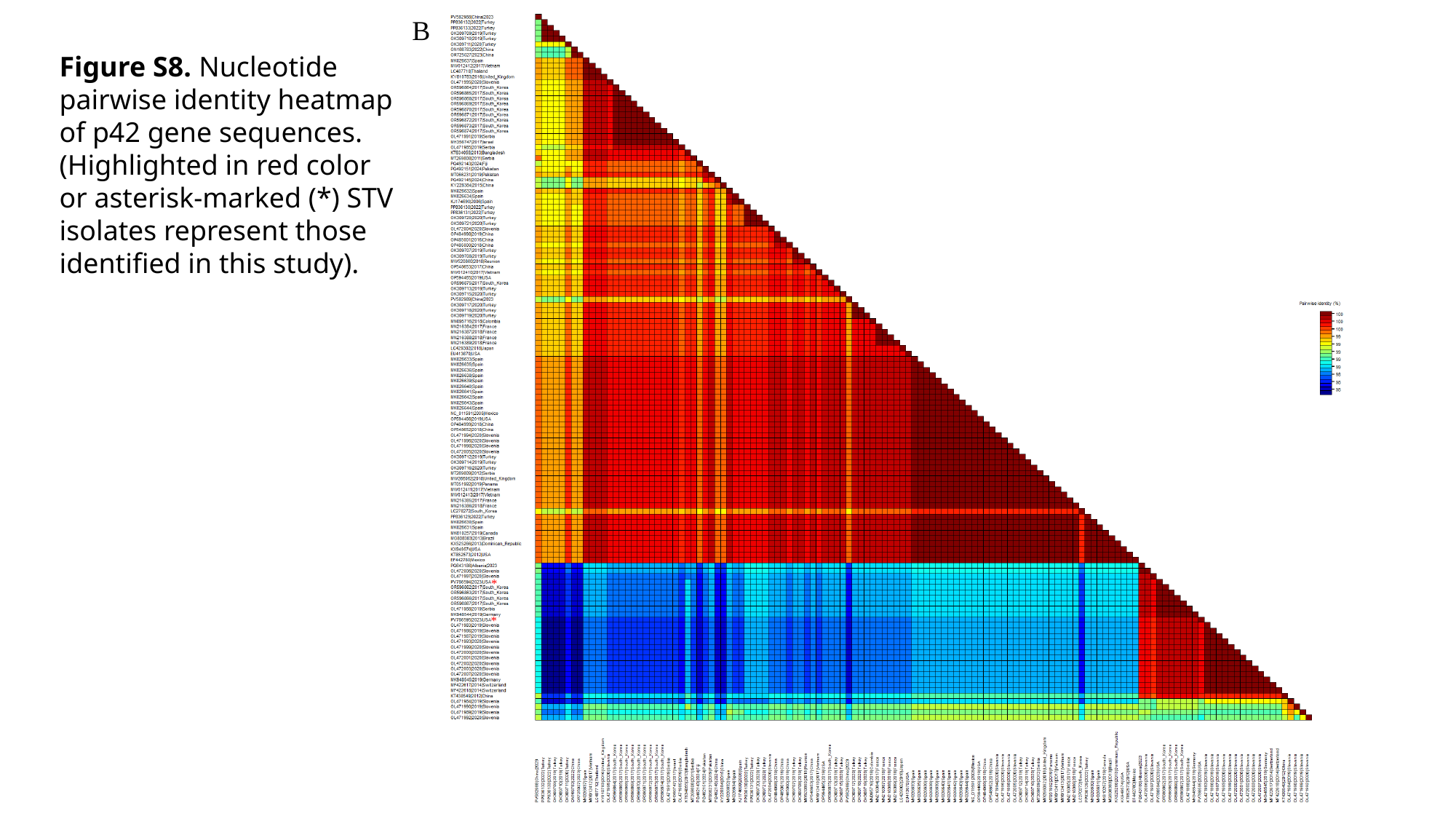

B
*
*
Figure S8. Nucleotide pairwise identity heatmap of p42 gene sequences. (Highlighted in red color or asterisk-marked (*) STV isolates represent those identified in this study).

## Slide 9
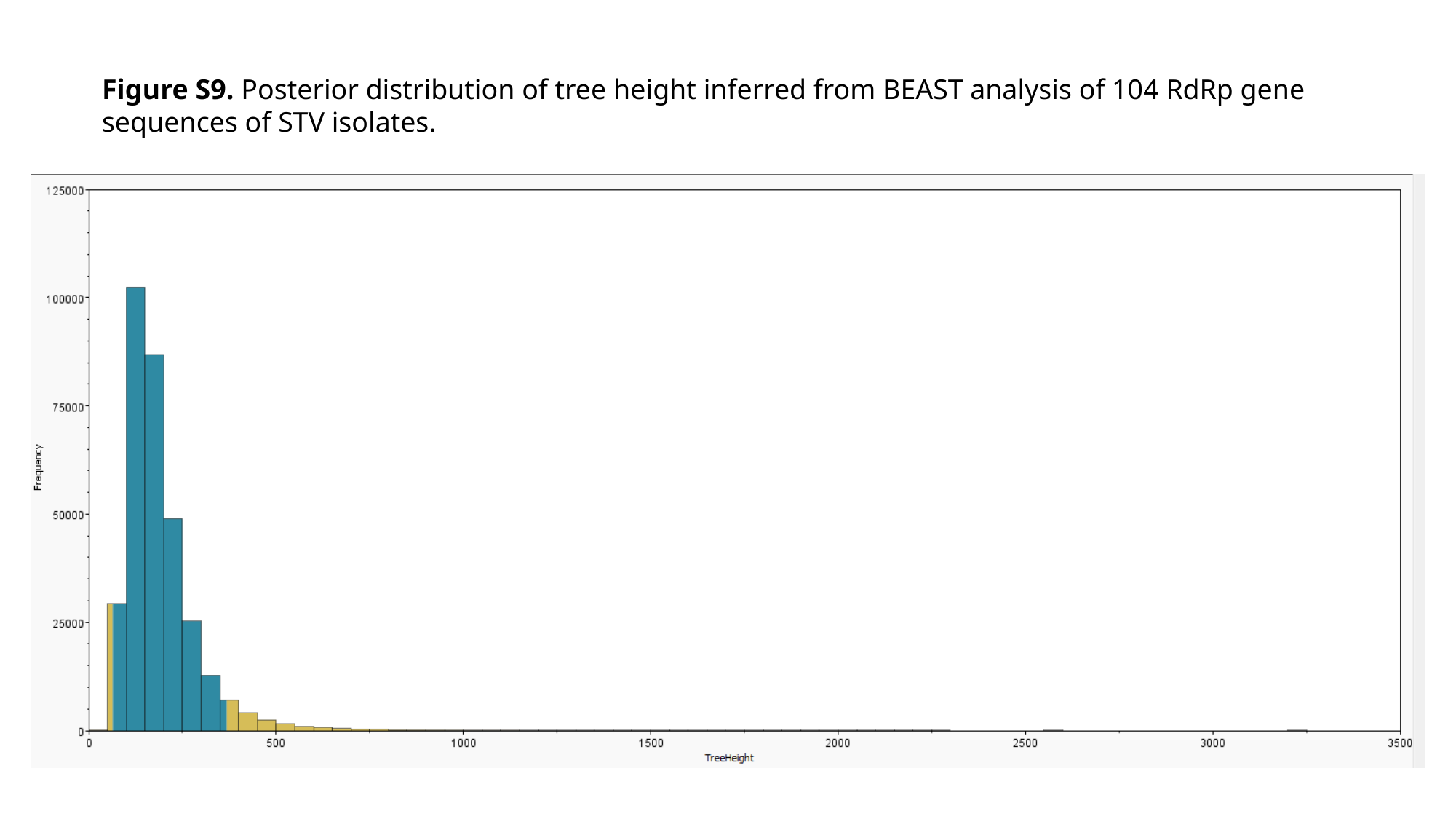

Figure S9. Posterior distribution of tree height inferred from BEAST analysis of 104 RdRp gene sequences of STV isolates.

## Slide 10
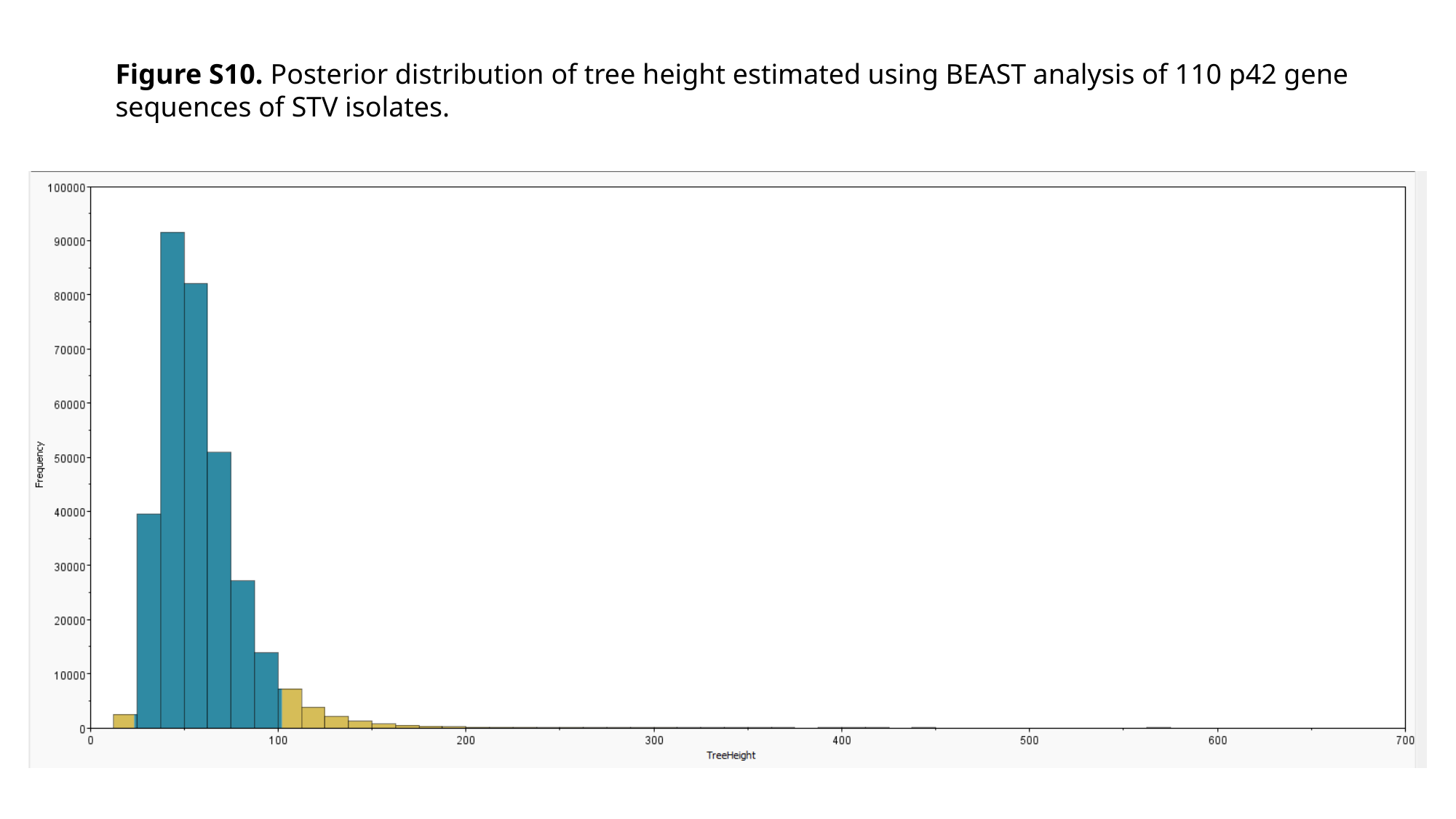

Figure S10. Posterior distribution of tree height estimated using BEAST analysis of 110 p42 gene sequences of STV isolates.

## Slide 11
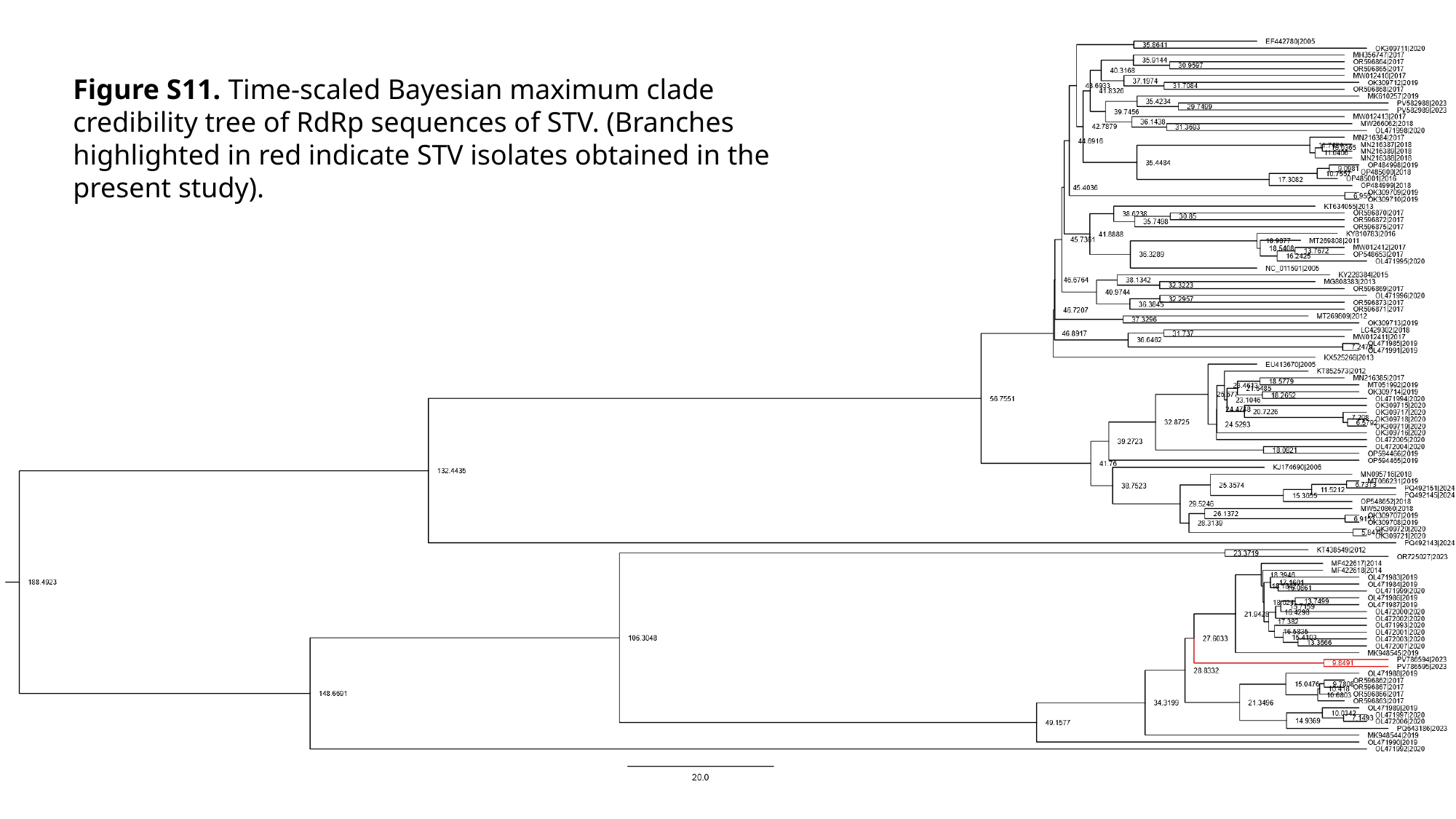

Figure S11. Time-scaled Bayesian maximum clade credibility tree of RdRp sequences of STV. (Branches highlighted in red indicate STV isolates obtained in the present study).

## Slide 12
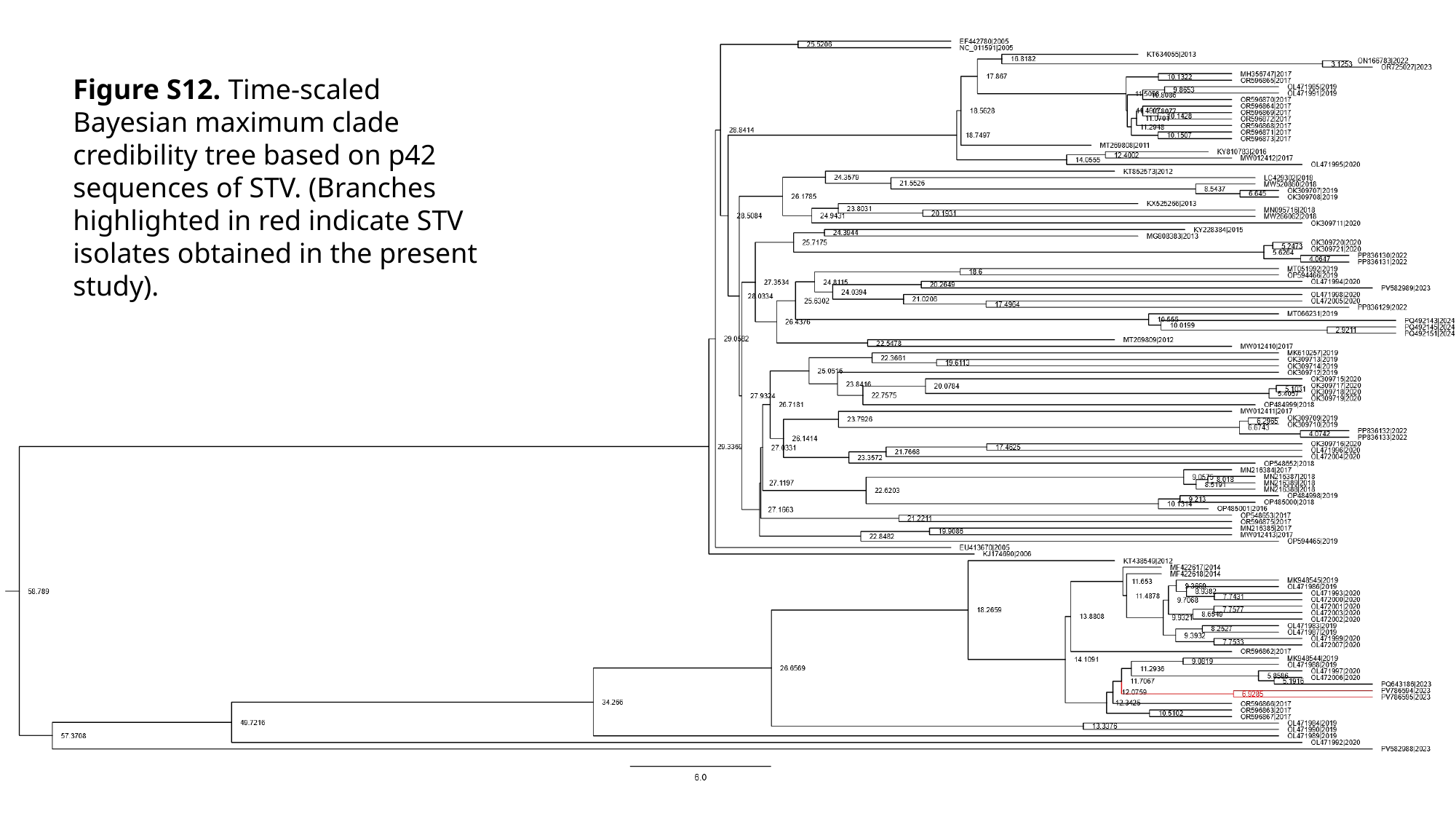

Figure S12. Time-scaled Bayesian maximum clade credibility tree based on p42 sequences of STV. (Branches highlighted in red indicate STV isolates obtained in the present study).

## Slide 13
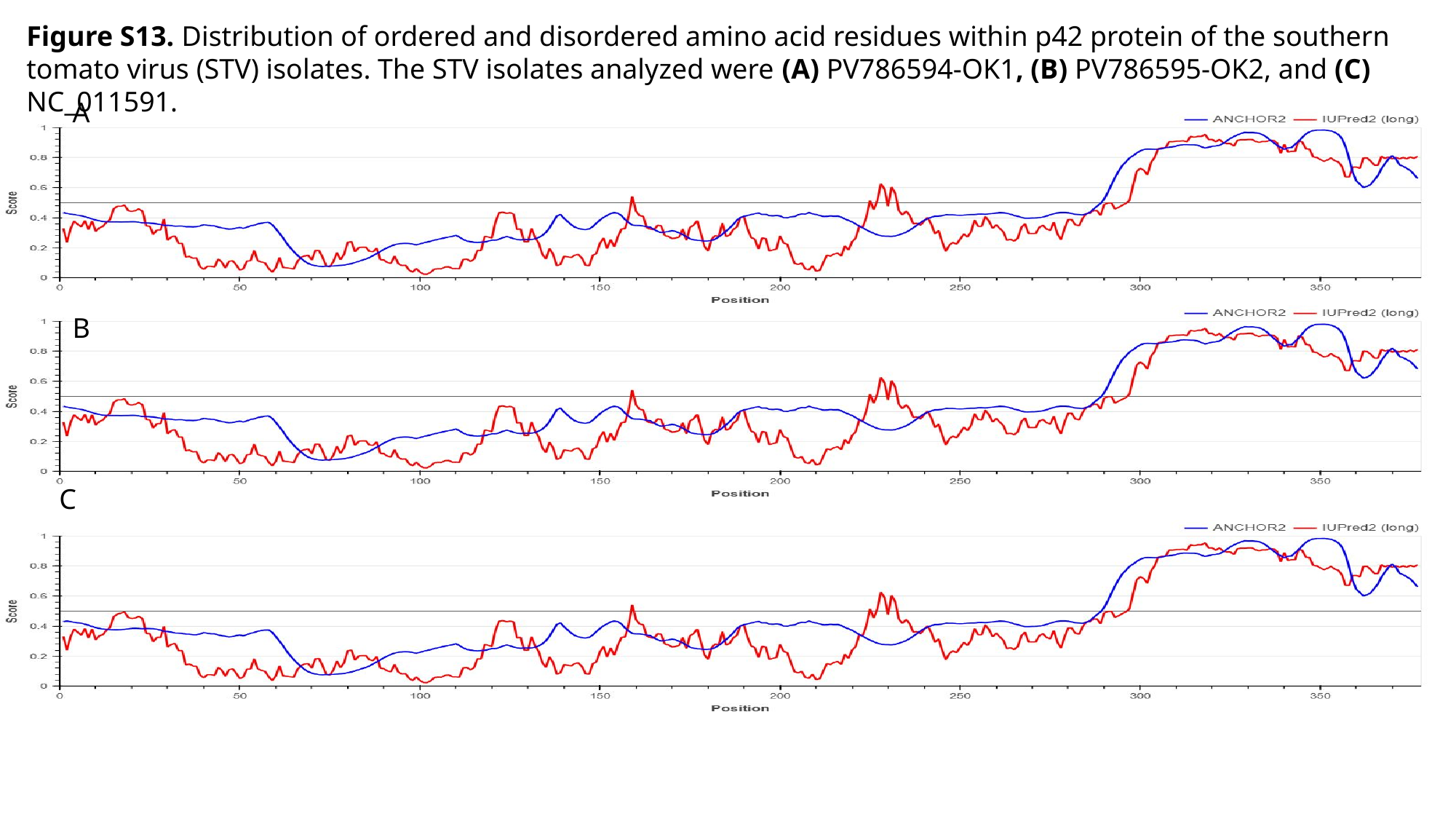

Figure S13. Distribution of ordered and disordered amino acid residues within p42 protein of the southern tomato virus (STV) isolates. The STV isolates analyzed were (A) PV786594-OK1, (B) PV786595-OK2, and (C) NC_011591.
A
B
C

## Slide 14
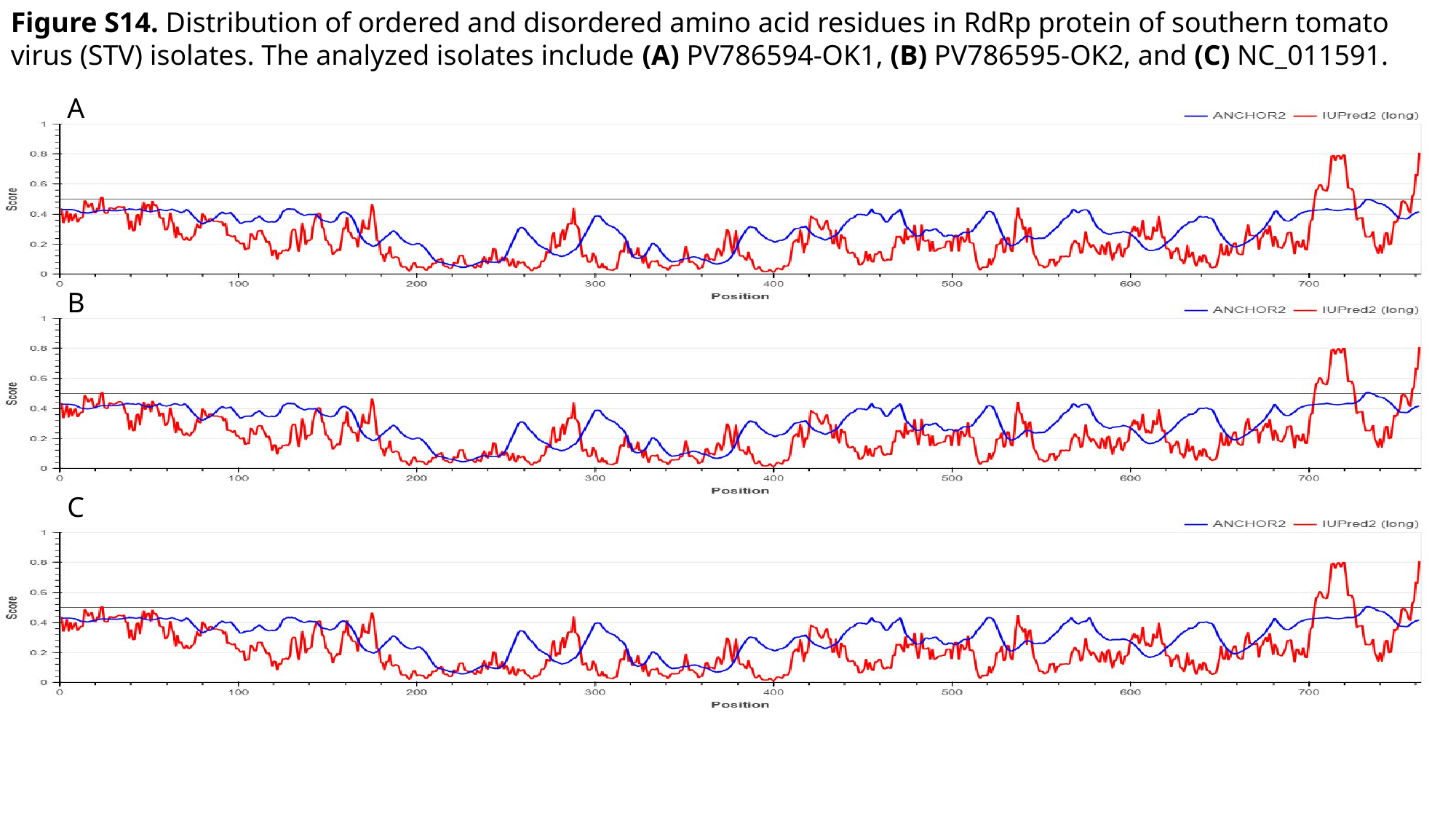

Figure S14. Distribution of ordered and disordered amino acid residues in RdRp protein of southern tomato virus (STV) isolates. The analyzed isolates include (A) PV786594-OK1, (B) PV786595-OK2, and (C) NC_011591.
A
B
C

## Slide 15
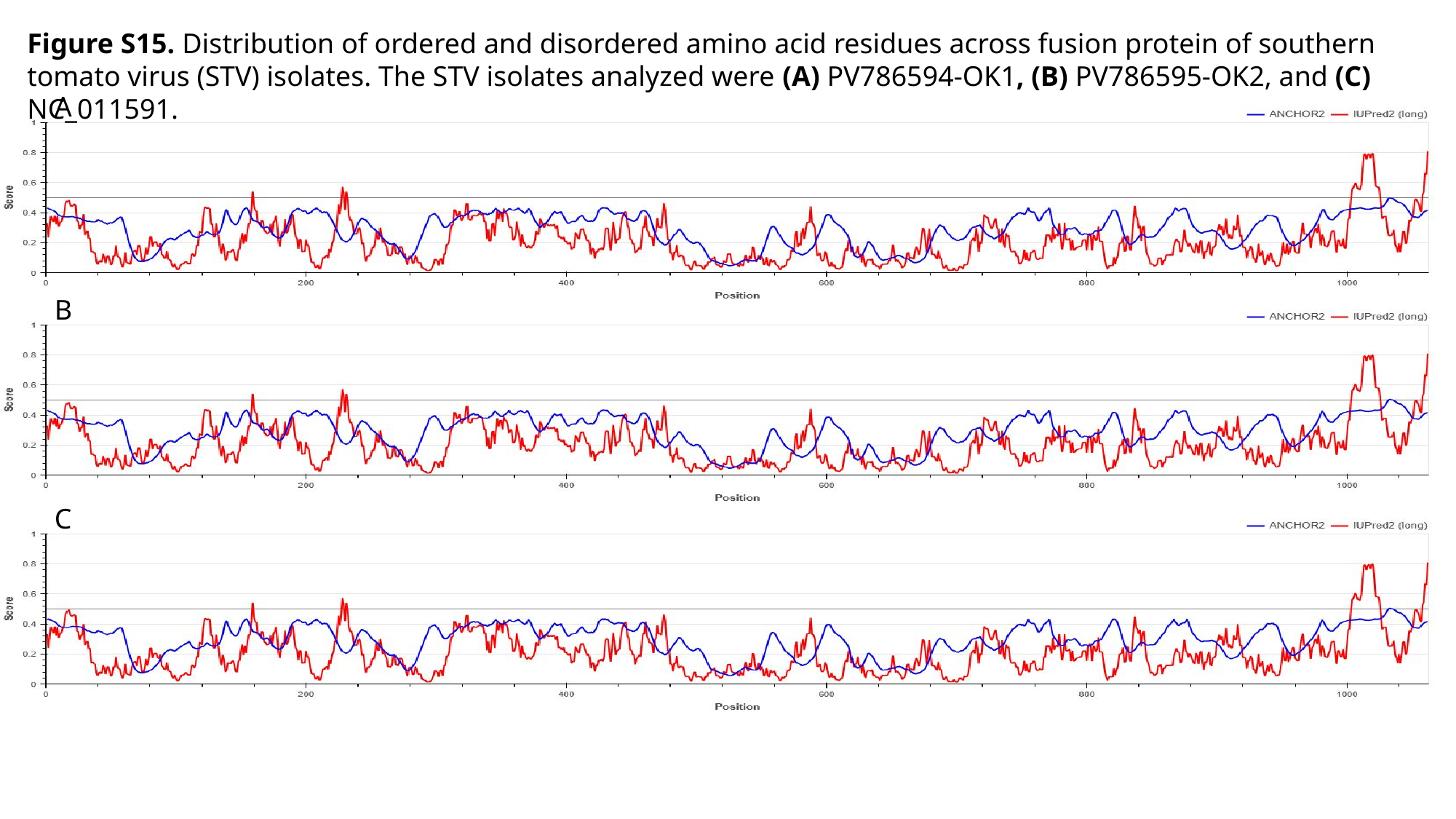

Figure S15. Distribution of ordered and disordered amino acid residues across fusion protein of southern tomato virus (STV) isolates. The STV isolates analyzed were (A) PV786594-OK1, (B) PV786595-OK2, and (C) NC_011591.
A
B
C
